# Unveiling the gut-heart potential connection: microbiota’s role in kawasaki disease and coronary artery lesions

**DOI:** 10.3389/fcimb.2025.1560083

**Published:** 2025-05-29

**Authors:** Qing Yang, Wei Tang, Jiayu Ren, Meng Li, Cuifen Zhao

**Affiliations:** Department of Pediatrics, Qilu Hospital of Shandong University, Jinan, China

**Keywords:** kawasaki disease, gut microbiota, coronary artery lesions, microbial biomarkers, SCFAs, opportunistic pathogens

## Abstract

**Background:**

Kawasaki disease (KD) is an acute systemic immune vasculitis predominantly affecting medium and small arteries, commonly observed in pediatric patients. It represents the most common form of acquired heart disease in this population. Emerging evidence suggests that gut microbiota can modulate the gut-vascular axis, influencing coronary artery lesions (CALs). This study aims to elucidate the potential association between gut microbiota, KD, and CALs, as well as identify bacterial biomarkers for CALs.

**Methods:**

We analyzed the gut microbiota composition of 60 children with KD (15 in the acute phase, 45 in the nonacute phase) and 30 healthy controls using alpha diversity indices and t-tests. Microbial biomarkers were identified through LEfSe to analyze the interplay between gut microbiota and CALs in KD during acute and non-acute phases.

**Results:**

In the acute phase, KD children exhibited decreased richness and diversity of gut microbiota, characterized by dysbiosis, particularly a reduction in short-chain fatty acid-producing bacteria and overgrowth of opportunistic pathogens. Thirteen genera showed statistically significant changes, including *Enterococcus, Bacteroides, Faecalibacterium, Agathobacter, Lachnospiraceae_NK4A136_group, Roseburia, Monoglobus*, etc. LEfSe analysis revealed enrichment of *Burkholderia-Caballeronia-Paraburkholderia, Hungatella, and Clostridium_innocuum group* in patients with concurrent CALs, while *Halomonas* was depleted. In the nonacute phase, gut microbiota diversity was similar to healthy controls, but *Streptococcus* was upregulated, while *Eubacterium_eligens_group* and *Erysipelotrichaceae_UCG_003* were downregulated. CALs were associated with reduced *Anaerostipes, Subdoligranulum, Roseburia, and Lachnospira*. LEfSe analysis also showed enrichment of *Coprobacillus, Paludicola, Lautropia, UCG-009, Acetanaerobacterium, Methylobacterium-Methylorubrum*, and *Alistipes* in patients with CALs.

**Conclusions:**

Our findings provide evidence of gut microbiota dysbiosis in KD children, suggesting its involvement in CALs development. It indicates that under the premise of standardized treatment during the acute phase of KD, it is plausible that microbiota-targeted strategies may alleviate CALs, thereby improving patient prognosis.

## 1 Introduction

Kawasaki disease(KD) is an acute systemic immune vasculitis with predominant involvement of the medium and small arteries. Coronary artery lesions (CALs), which occur in approximately 15~25% of untreated patients, have emerged as the most common form of acquired heart disease in patients in developed countries (McCrindle et al., 2017). This study is a cross-sectional study. Participants come from outpatient follow-up patients and ward patients at the sampling time point. In order to control the bias caused by time factors as much as possible, the sample size is limited. In view of the high risk of myocardial infarction in KD patients with CALs 2–3 months before the course of the disease. The patients with KD in the non-acute phase were evaluated at 3 months after discharge from the outpatient department of this study group. At present, the pathogenesis of KD is still unclear, and studies on the etiology of KD have focused on susceptibility genes, infectious factors, and immunologic factors. The gut microbiota, defined as the bacteria inhabit the gastrointestinal tract, has been linked to several diseases. Microbial diversity plays a key role in the maintenance of intestinal homeostasis and the development of the intestinal mucosal immune system. Recent research has indicated that dysbiosis of the gut microbiota is linked with the development of KD in genetically susceptible children (Yang et al., 2025).

Current evidence has revealed that the diversity of the gut microbiota in patients with KD was significantly reduced in comparison to that of the healthy controls, with a notable reduction in the short-chain fatty acids (SCFAs) -producing microbiota. The relative abundance of *Enterococcus, Acinetobacter, Helicobacter, Lactococcus, Staphylococcus, Streptococcus, Butyromonas, Fusobacterium*, and *Shigella* in the acute stage of KD was significantly increased, while *Bacteroides* and *Dorea* were decreased. Recovery of beneficial bacteria may potentially correlate with remission in KD patients, especially *Ruminococcus, Bacillus vulgaris, Bifidobacterium, Lactobacillus, and Roseburia*. The relative abundance of the *Ruminococcus gnavus* group was higher and *Blautia* was lower in children with a history of KD (Kinumaki et al., 2015; Chen et al., 2020; Khan et al., 2020; Shen et al., 2020; Teramoto et al., 2023; Wang et al., 2023; Zeng et al., 2023). Kaneko used high-performance liquid chromatography to quantify fecal organic acids (Kaneko et al., 2020). The results showed a significant decrease in fecal butyrate concentration in the KD group, whereas the levels of acetate, lactate, and propionate remained comparable between the two groups.

So far, there is few study on the correlation between KD with CALs and gut microbiota. In this study, 16S-rRNA gene amplicon was used to detect and analyze the gut microbiota of children with KD and healthy children. We will analyze the changes of gut microbiota in children with KD at different stages of the disease and predict its biological function. At the same time, the correlation between CALs and the composition and distribution of intestinal flora was studied, hoping to provide a basis for finding ways to improve the prognosis of KD with CALs.

## 2 Materials and methods

### 2.1 Study subjects

Children with KD who were admitted to Qilu Hospital of Shandong University from September 2023 to February 2024 were selected as study subjects. Healthy children who were examined at the Children’s Health Care Clinic served as the control group.

Inclusion criteria: The diagnostic criteria of KD, JCS/JSCS 2020 Guideline on Diagnosis and Management of Cardiovascular Sequelae in Kawasaki Disease, were met (Fukazawa et al., 2020). That is, the child has a fever for >5d and meets several major clinical features: ① bilateral bulbar conjunctival congestion; ② changes in lips and mouth: dry red lips, strawberry tongue, diffuse congestion of the mucous membrane of the oropharynx; ③ rashes, including isolated cicatricial erythema; ④ changes at the end of the limbs: redness and swelling of the hands and feet in the acute phase, and peri-nail desquamation in the recovery phase; ⑤ non-suppurative cervical lymph node enlargement.

Exclusion criteria: ① application of proton pump inhibitors, probiotics, or prebiotics in the last 1 month; ② combination of other vasculitis, immune diseases, and chronic gastrointestinal diseases. In view of the poor appetite of children with KD in the acute phase, the samples of children in the non-acute phase were collected retrospectively, and the children took aspirin. Due to the responsibility for the life and health of the children and the actual limitations, it is difficult for us to set up a control group with KD and the same diet as the normal people without drug intervention, so it is difficult to achieve diet control.

A total of 62 cases of children with KD (2 cases were discarded due to irregularities in sample retention) and 30 cases of healthy children were included. Stool specimens were collected from all the subjects, 16S-rRNA sequencing was performed to analyze the intestinal flora, and the clinical data of KD patients (gender, age, duration of the disease, cardiac ultrasound, and medication) were collected for the correlation analysis of the gut microbiota and the clinical indexes. CAL is defined as coronary artery internal diameters that were greater than 3 mm in the main trunk of the coronary arteries in children younger than 5 years of age, greater than 4 mm in the main trunk of the coronary arteries in children older than 5 years of age, or localized internal diameters that were more than 1.5 times larger than those of neighboring locations, or coronary artery internal diameter Z-values≥2.0.

### 2.2 Fecal samples collection

Fresh stool samples were collected in sterile plastic tubes from children with KD at the time of their first bowel movement after admission/outpatient follow-up. Stool specimens were collected from healthy controls on the day of the visit. All stool samples were immediately transferred to the laboratory in an ice box, stored at - 80°C within 30 minutes and uniformly sent to the laboratory for 16S rRNA sequencing.

### 2.3 Extraction of genome DNA

Genomic DNA was extracted from samples using the Hexadecyl trimethyl ammonium bromide (CTAB) method. The concentration and purity of the DNA were assessed by running a 1% agarose gel electrophoresis. Based on the determined concentration, the DNA was then diluted to a final concentration of 1 ng/µL using sterile water.

### 2.4 Amplicon generation

The V3-V4 regions of the 16S rRNA genes were amplified using the primers 341F (CCTAYGGGRBGCASCAG) and 806R (GGACTACNNGGGTATCTAAT), which included barcodes. Each PCR reaction consisted of 15 µL of Phusion^®^ High-Fidelity PCR Master Mix (New England Biolabs), 2 µM of each forward and reverse primer, and approximately 10ng of template DNA. Thermal cycling conditions included an initial denaturation step at 98°C for 1 minute, followed by 30 cycles of denaturation at 98°C for 10 seconds, annealing at 50°C for 30 seconds, and elongation at 72°C for 30 seconds, with a final extension step at 72°C for 5 minutes.

### 2.5 PCR products quantification and qualification

To evaluate the PCR products, an equal volume of 1X loading buffer (containing SYBR Green) was mixed with the PCR products, and the mixture was subjected to electrophoresis on 2% agarose gel for visualization. The PCR products were then combined in equimolar ratios. The resulting mixture of PCR products was purified using the Qiagen Gel Extraction Kit (Qiagen, Germany).

### 2.6 Library preparation and sequencing

Sequencing libraries were constructed using the TruSeq^®^ DNA PCR-Free Sample Preparation Kit (Illumina, San Diego, CA, USA), following the manufacturer’s protocols, and index codes were incorporated into the libraries. The quality of the libraries was assessed using the Qubit^®^ 2.0 Fluorometer (Thermo Scientific) and the Agilent Bioanalyzer 2100 system. Finally, the libraries were sequenced on an Illumina NovaSeq platform, generating 250 bp paired-end reads.

### 2.7 Bioinformatics analysis

The raw data acquired from the Illumina Nova sequencing platform underwent preprocessing, including division, removal of primer sequences, splicing of paired-end reads, and filtering and truncation based on tag quality and length to obtain the final set of effective tags. An operational taxonomic unit (OTU) table was constructed using these valid sequences. To investigate the phylogenetic relationships among different OTUs and the variations in dominant species across groups, multiple sequence alignments were performed using MUSCLE software (Version 3.8.31, http://www.drive5.com/muscle/). The alpha diversity differences between groups were evaluated using Student’s t-test. A rarefaction curve was utilized to assess the saturation of microbiome sequencing or sample size. Species accumulation curves were employed to estimate species richness in a given area, reflecting the impact of sample number on species diversity. Linear discriminant analysis effect size (LEfSe) method, combined with Kruskal-Wallis rank-sum tests and linear discriminant analyses (LDAs), was applied to identify differentially abundant taxa and discriminate metagenomic biomarkers. An alpha significance level of 0.05 and an effect size threshold of 2 or 2.5 were set for identifying significant microbial biomarkers. The functional composition of microbiota communities was predicted using Phylogenetic Investigation of Communities by Reconstruction of Unobserved States (PICRUSt) based on 16S rRNA gene data, resulting in a functional abundance spectrum of the Kyoto Encyclopedia of Genes and Genomes (KEGG) orthologs.

### 2.8 Statistical analysis

Quantitative data are presented as mean ± standard deviation (mean ± SD). Draw the sample dilution curve to evaluate whether the sequencing depth is appropriate. Pairwise comparison is used for inter group analysis, lefse analysis is used to explain the difference of species composition between groups, and the description of LDA cut-off value and P value is detailed in the comparative analysis of each group. Comparisons between the two groups were conducted using Student’s t-test. To facilitate intuitive visualization of the data, various graphical representations including Venn diagrams, box plots, bar charts, Sankey diagrams, and cluster maps were employed. A p-value < 0.05 was considered statistically significant.

## 3 Results

**Figure 1:**
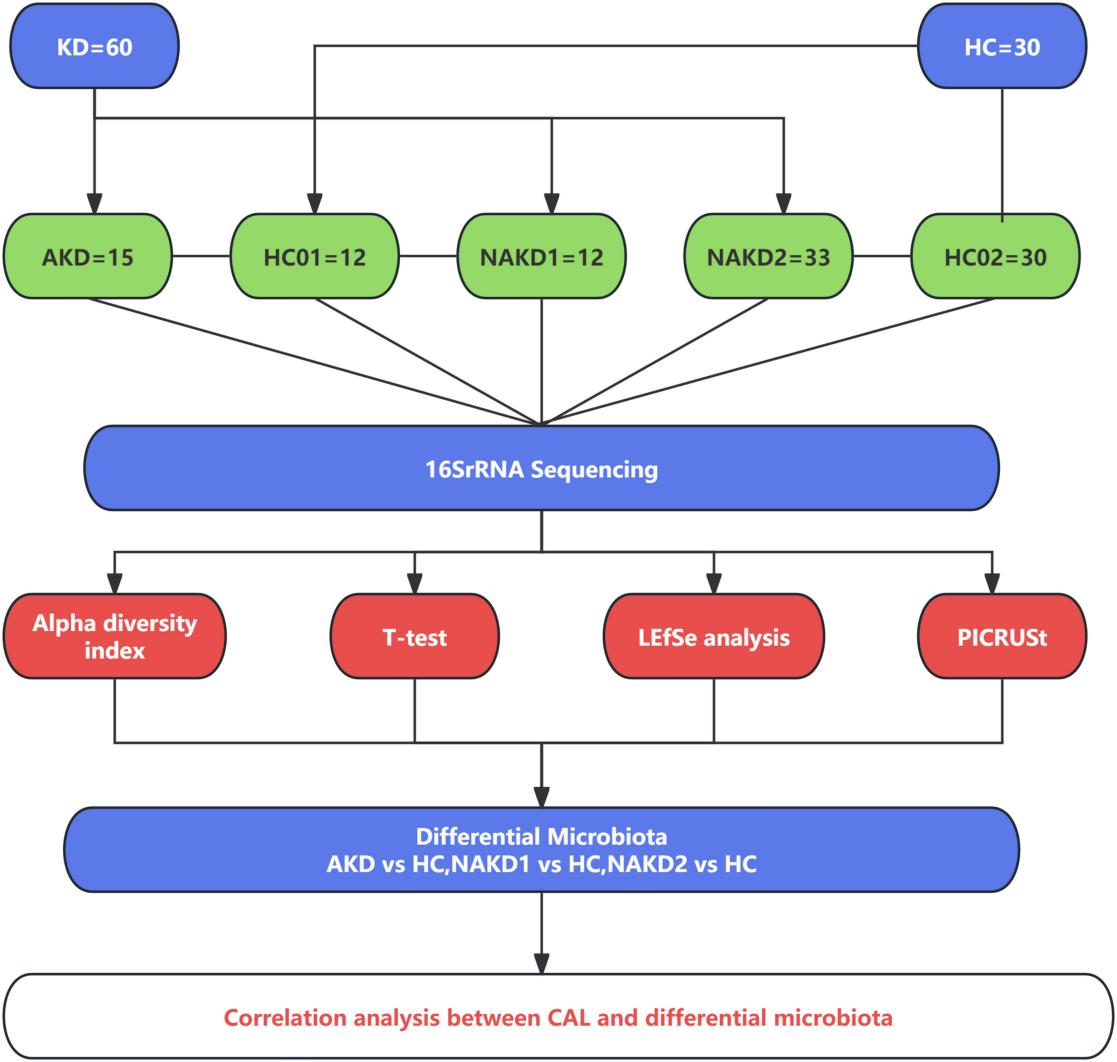
Overview of the study design.

The patients with Kawasaki disease in the non-acute phase were evaluated at 3 months after discharge from the outpatient department of this study group (Jone et al., 2024).The KD children in this study were divided into acute phase (AKD) and non-acute phase (NAKD) according to the course of the disease, and the acute phase was defined as the hospitalization duration. The non-acute phase with the course of disease less than 3 months was defined as the NAKD1 group, and the course of disease more than 3 months was defined as the NAKD2 group. Age - and sex-matched healthy controls (HC) were used as the control group, and a total of 30 samples were retained, labeled as HC01 and HC02 (HC02 included HC01). The control group for AKD and NAKD1 was HC01, and the control group for NAKD2 was HC02 (**Figure 1**).

### 3.1 Statistical analysis

Fifteen AKD patients [average age (2.42 ± 1.63) years, 7 males and 8 females] were enrolled in this study. Eight patients had CAL (AKDCAL), and seven patients had no CAL (AKDNCAL). Forty-five NAKD patients [average age (3.32 ± 1.85) years, 29 males and 16 females] were involved. There were 12 patients in the NAKD1 group, with an average age of 2.01 ± 0.94 years, 8 males and 4 females, 4 patients with CAL, and 8 patients without. All patients took aspirin. There were 33 patients in the NAKD2 group, with an average age of 3.80 ± 1.88 years, 21 males and 12 females. Among them, 9 patients with CAL (NAKDCAL) took aspirin, and 24 patients without CAL (NAKDNCAL) did not. There were 15 patients in the HC01 group, including 7 males and 8 females, with an average age of (2.48 ± 1.47) years. The HC02 group involved 30 patients, including 14 males and 16 females, with an average age of (3.96 ± 1.99) years (**Supplementary Tables 1**–**3**).

### 3.2 Overview of all samples sequencing

Following a quality control process on the sequences, a total of 90 fecal specimens were successfully sequenced, containing 60 children with KD and 30 healthy children. Dilution curves flattened out in all samples (**Supplementary Figure S1**), and species accumulation boxplot curves were asymptotically stable (**Supplementary Figure S2**), indicating that the sequencing depth was sufficient and could accurately represent the gut microbiota composition. The relative abundance of gut microbiota in phylum and genus for each sample is shown in the following (**Supplementary Figures S3**, **S4**).

### 3.3 AKD group vs HC01 group

#### 3.3.1 Differences in gut microbiota diversity and microbiota composition between the AKD group and HC01 group

**Figure 2:**
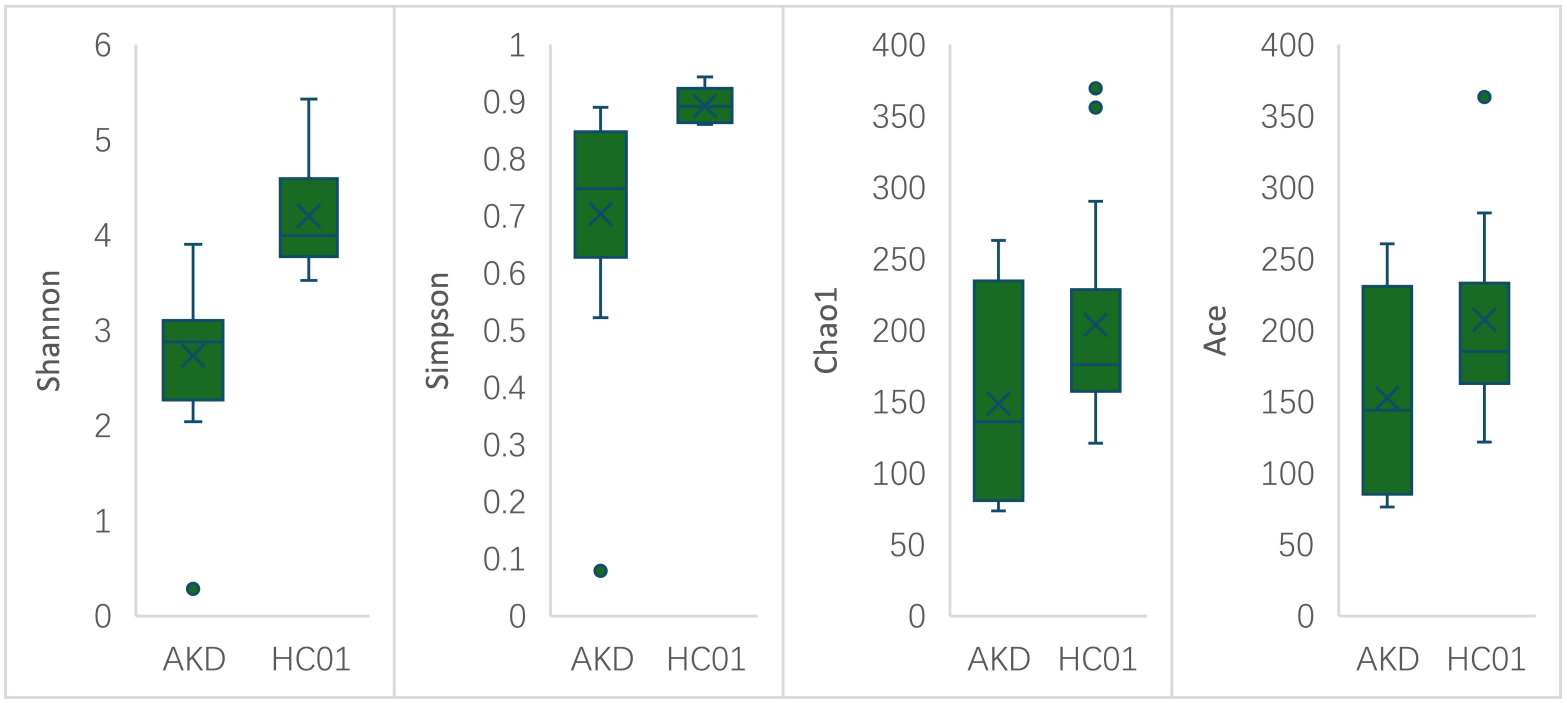
Alpha diversity index of AKD and HC01.

**Figure 3:**
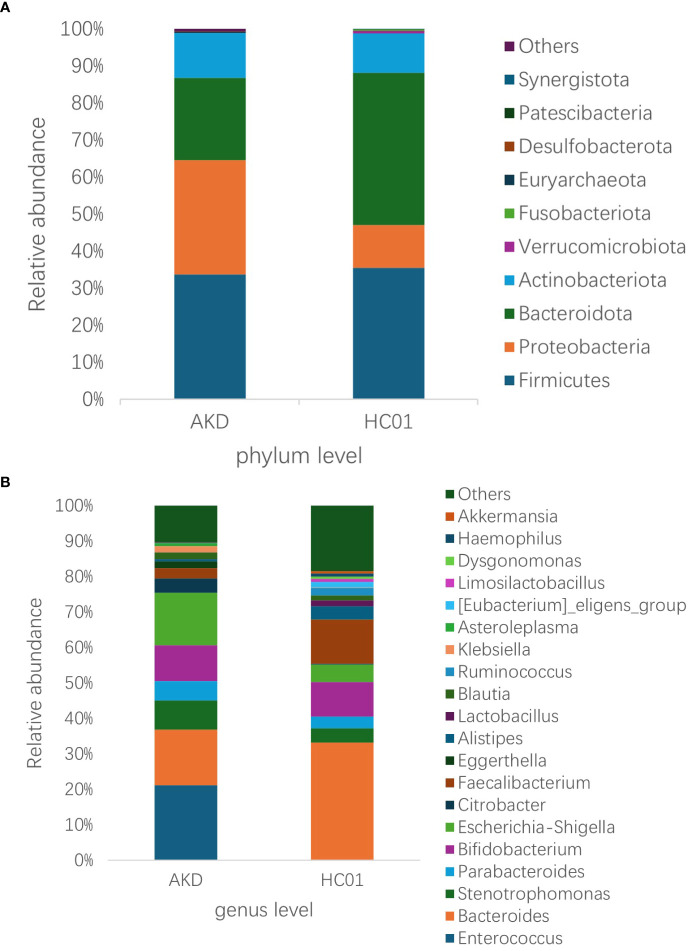
Relative abundance of species at the **(A)** phylum and **(B)** genus level for AKD and HC01.

**Figure 4:**
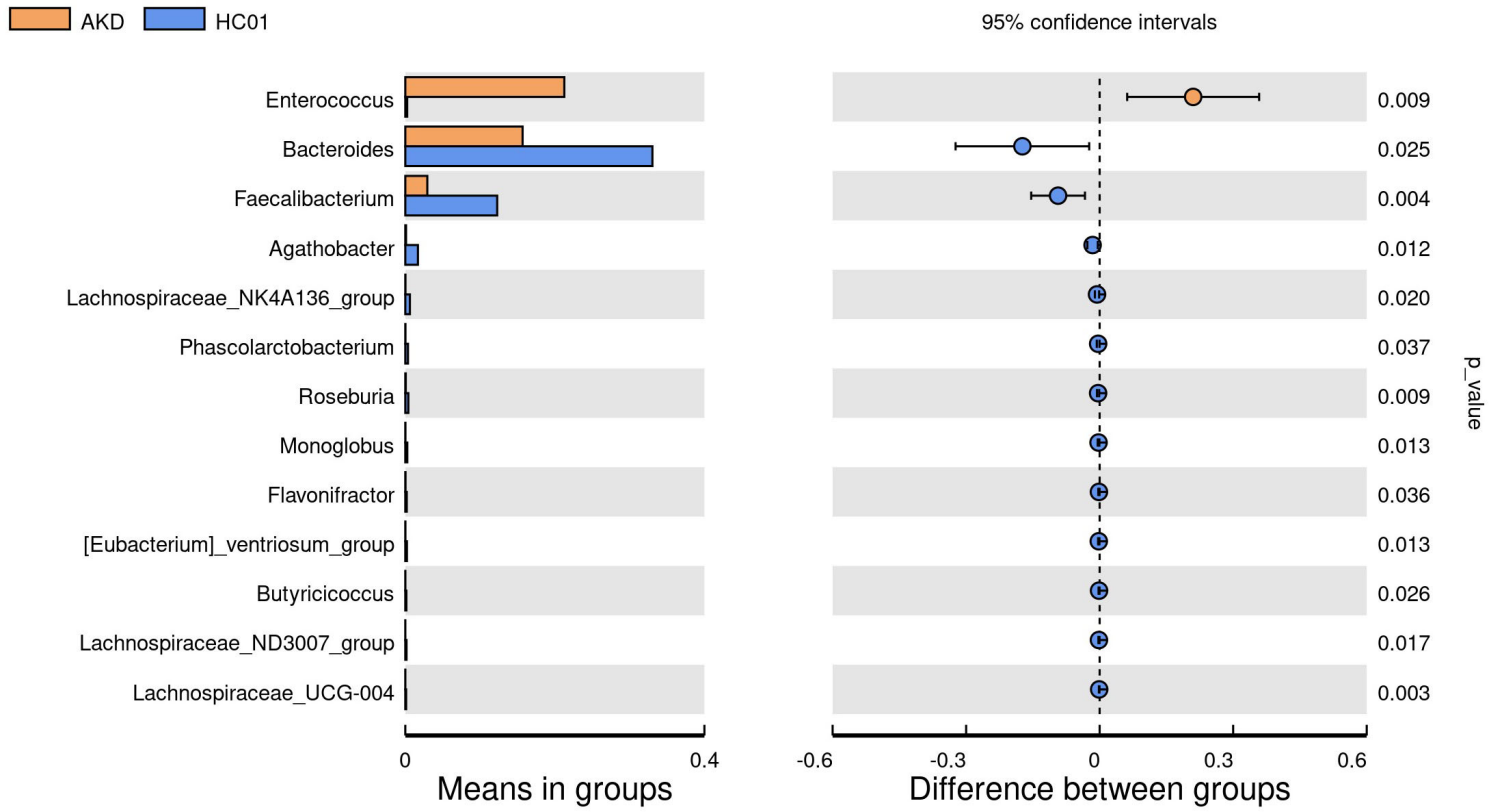
T-test at genus level between AKD and HC01.

There was no statistically significant difference in age and sex composition ratio between AKD and HC01 groups. We obtained 588 OTUs from fecal specimens of AKD, which was a decrease in the number of OTUs as compared to 661 OTUs in HC01, and the Veen diagram demonstrated the characteristic sequence of each group (**Supplementary Figure S5**). Alpha diversity index was used to assess the abundance and diversity of the microbiota. Significantly lower Shannon index (p=0.000), Simpson index (p=0.030), Chao1 index (p=0.047), and Ace index (p=0.046) of intestinal flora were observed in the AKD (**Figure 2**). Which revealed a significant difference in microbial richness and diversity between KD children and healthy children, indicating a reduction in the richness and diversity of gut microbiota in pediatric KD. The human gut microbiota was categorized as consisting of Firmicutes, Proteobacteria, Bacteroidota, and Actinobacteriota at the phylum level. AKD was mainly composed of Firmicutes (33.7%), Proteobacteria (30.9%), Bacteroidota (22.1%), and Actinobacteriota (12.2%). HC01 was composed of Firmicutes (35.5%), Proteobacteria (11.6%), Bacteroidota (41.1%), and Actinobacteriota (10.7%) (**Figure 3A**). At the phylum level, Proteobacteria were significantly increased (p=0.024), and Bacteroidota (p=0.033) decreased in AKD. We enumerated each of the two groups of species whose relative abundance exceeded 10% at the genus level. The AKD group included *Enterococcus* (21.3%), *Bacteroides* (15.7%), *Escherichia* (14.9%) and *Bifidobacterium* (10.1%). The HC01 group involved *Bacteroides* (33.0%), and *Faecalibacterium* (12.3%) (**Figure 3B**). At the genus level, *Enterococcus* (p=0.009) and *Escherichia-Shigella* (p=0.052) were increased in the AKD group, while *Bacteroides* (p=0.025) and *Faecalibacterium* (p=0.004) were decreased. The Sankey diagrams were able to visualize more clearly the changes in the two groups of major bacterial groups (**Supplementary Figures S6A, B**). A t-test was performed to explore all the different strains in the two groups at the genus level. A total of 13 genera of bacteria with statistically significant (p < 0.05) changes, such as *Enterococcus, Bacteroides, Faecalibacterium, Agathobacter, Lachnospiraceae_NK4A136_ group, Roseburia, Monoglobus*, etc. (**Figure 4**). For species with low frequencies, i.e., the sum of their respective frequencies (absolute abundance of species) within the two groups was less than the number of samples in the group, both cluster plots (**Supplementary Figure S7**) and Boxplot plots (**Supplementary Figure S8**) in the results of the Meta-stat analyses showed that there was a decrease in the abundance of a variety of species in the AKD group.

#### 3.3.2 Potential bacterial biomarkers of AKD patients

**Figure 5:**
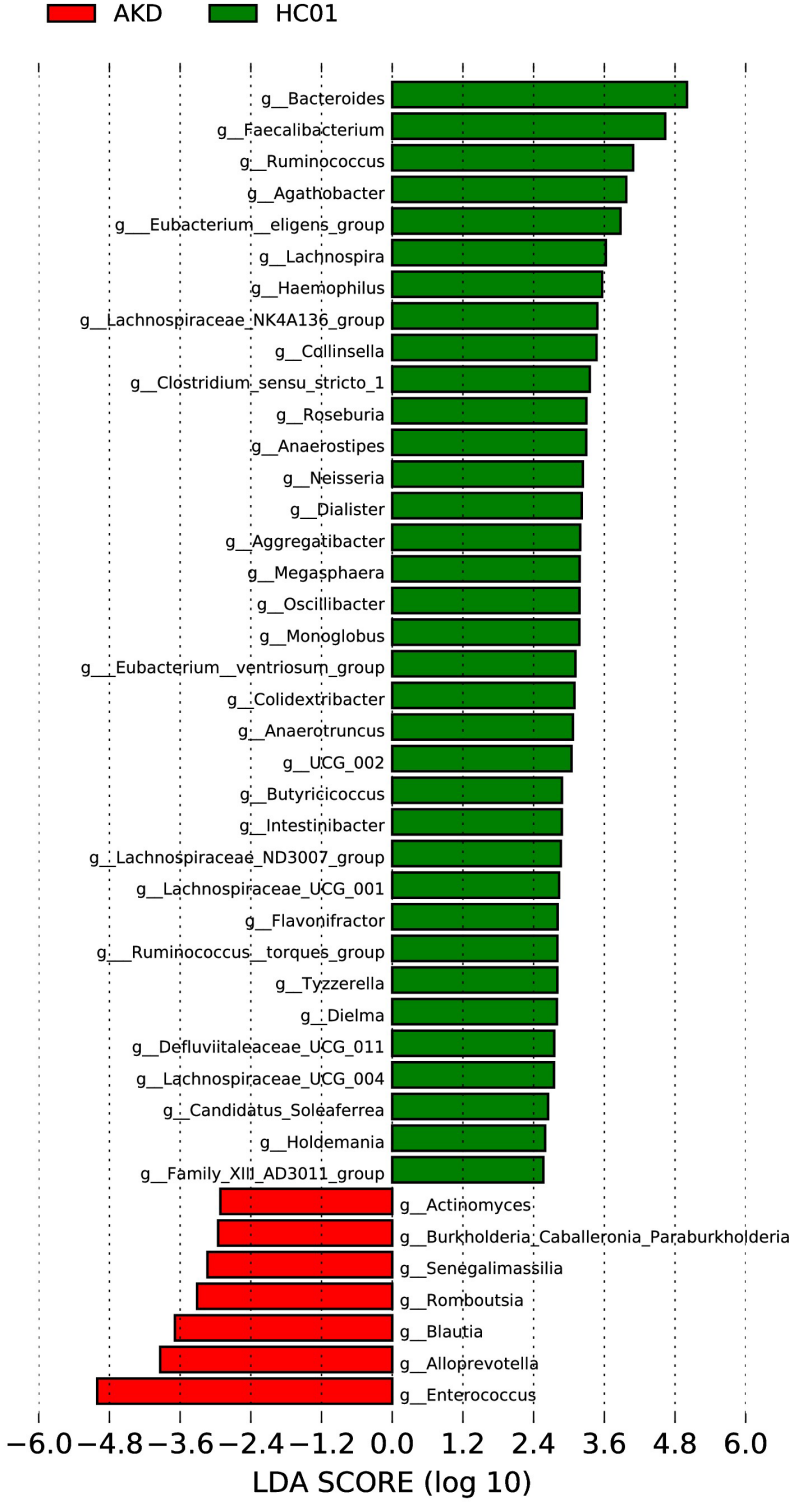
LEfSe of AKD and HC01 at genus level.

The LEfSe analysis at the genus level (LDA score [log10] >2.5, p < 0.05) was performed to identify potential bacterial biomarkers associated with KD. Several microbiota were found to be significantly enriched in KD patients, including *Enterococcus, Alloprevotella, Blautia, Romboutsia, Senegalimassilia, Burkholderia-Caballeronia-Paraburkholderia*, and *Actinomyces*. Conversely, several microbiota known to be involved in SCFA production exhibited a significant decrease in abundance among KD patients, including *Bacteroides, Faecalibacterium, Ruminococcus, Lachnospiraceae, Eubacterium, Clostridium*, and *Prevotella* (**Figure 5**).

#### 3.3.3 Functional capability analysis

The functional gene composition of microbiota in fecal samples was analyzed by the PICRUSt (student’s t-test) to differentiate the normal biological function between AKD patients and healthy controls. For KEGG level 1 (**Supplementary Figure S9A**), the AKD group exhibited elevated activity in metabolism, environmental information processing, and human diseases, while the HC01 group demonstrated heightened activity in genetic information processing and organismal systems. At the KEGG level 2 level (**Supplementary Figure S9B**), membrane transport, metabolism, xenobiotics biodegradation and metabolism, signal transduction, and infectious diseases were found to be significantly higher in the AKD group. Conversely, the HC01 group exhibited higher levels in amino acid metabolism, replication and repair, metabolism of cofactors and vitamins, enzyme families, biosynthesis of other secondary metabolites, cell growth and death, endocrine system, environmental adaptation, nervous system, and metabolic diseases. Furthermore, a multitude of KEGG pathways manifested statistically significant differences at level 3 (**Supplementary Figure S9C**). Of particular interest are the two-component systems, secretion systems, and phosphotransferase systems (PTS), which were found to be significantly elevated in the AKD group.

#### 3.3.4 Relationship between CAL and gut microbiota changes in AKD patients

To investigate the correlation between CAL and the shifts in gut microbiota in the AKD, a statistical analysis was conducted between AKDCAL and AKDNCAL. The analysis yielded 400 OUTs from fecal samples in the AKDCAL group, which was less than the 478 OTUs observed in the AKDNCAL group. However, the alpha diversity between these two groups did not demonstrate a statistically significant difference. The predominant phyla in the AKDCAL sample included Bacteroidota (33.8%), Firmicutes (27.3%), Proteobacteria (23.3%), and Actinobacteriota (15.0%). In contrast, the predominant phyla in the AKDNCAL sample included Firmicutes (41.0%), Proteobacteria (39.7%), Actinobacteriota (9.0%), and Bacteroidota (8.8%). At the genus level, *Enterococcus* (13.2%), *Bacteroides* (23.9%), *Escherichia* (13.4%), and *Bifidobacterium* (14.8%) were identified as the most prevalent pathogens in AKDCAL. *Enterococcus* (30.5%) and *Escherichia coli* (16.5%) were identified as the predominant pathogens in AKDNCAL. While there were some differences in species composition between the two, no statistically significant strains were identified at each level of species classification.

**Figure 6:**
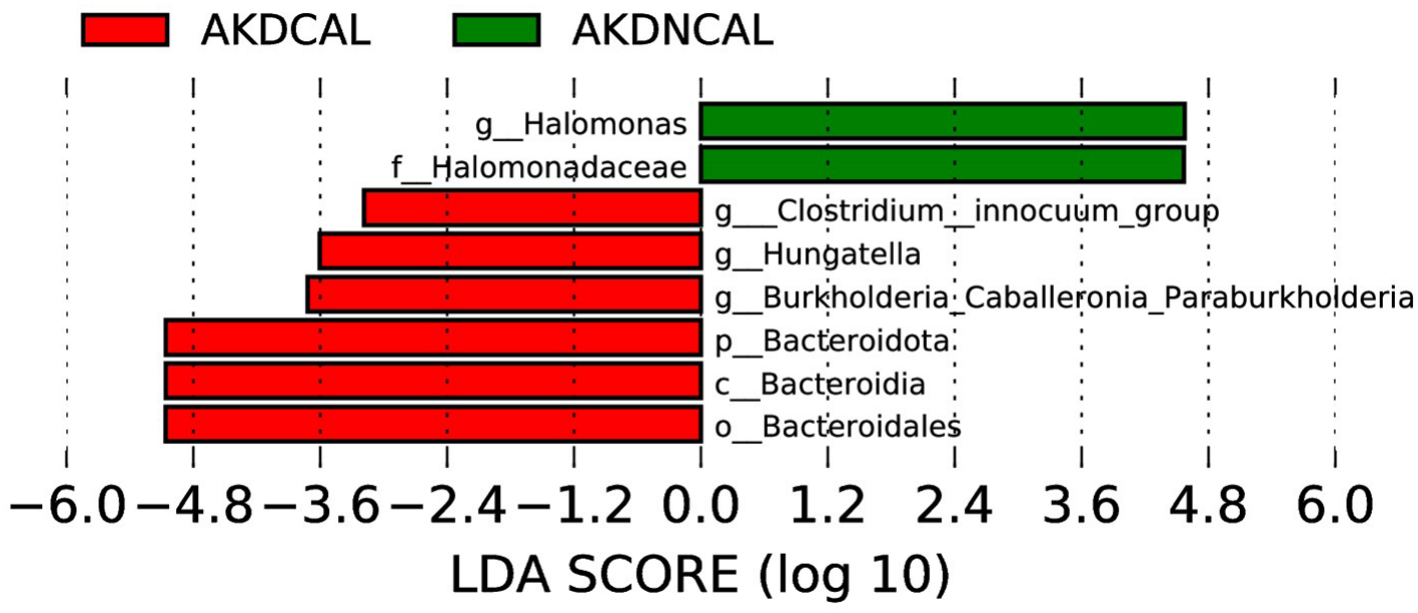
LEfSe of AKDCAL and AKDNCAL.

The LEfSe analysis (LDA score (>log10) >2.0, p < 0.05) revealed that *Burkholderia-Caballeronia-Paraburkholderia, Hungatella*, and *Clostridium-inoculum group* were enriched in AKDCAL, whereas *Halomonas* increased in AKDNCAL at the genus level (**Figure 6**). We used PICRUSt to differentiate the normal biological function between AKDCAL and AKDNCAL. At KEGG level 1 (**Supplementary Figure S10A**), the AKDCAL group exhibited higher metabolic activity, while the AKDNCAL group demonstrated higher environmental information processing (*P* < 0.05). At KEGG level 2 (**Supplementary Figure S10B**), membrane transport was significantly higher in the AKDNCAL group. At KEGG level 3 (**Supplementary Figure S10C**), the AKDCAL group exhibited heightened levels of peptidases, amino acid-related enzymes, DNA replication proteins, cysteine and methionine metabolism, alanine, aspartate, and glutamate metabolism, transcription machinery, and histidine metabolism. Notably, the AKDNCAL group demonstrated a significant increase in the levels of the transcription factor.

### 3.4 NAKD1 group vs HC01 group

#### 3.4.1 Differences in gut microbiota composition between NAKD1 and HC01

**Figure 7:**
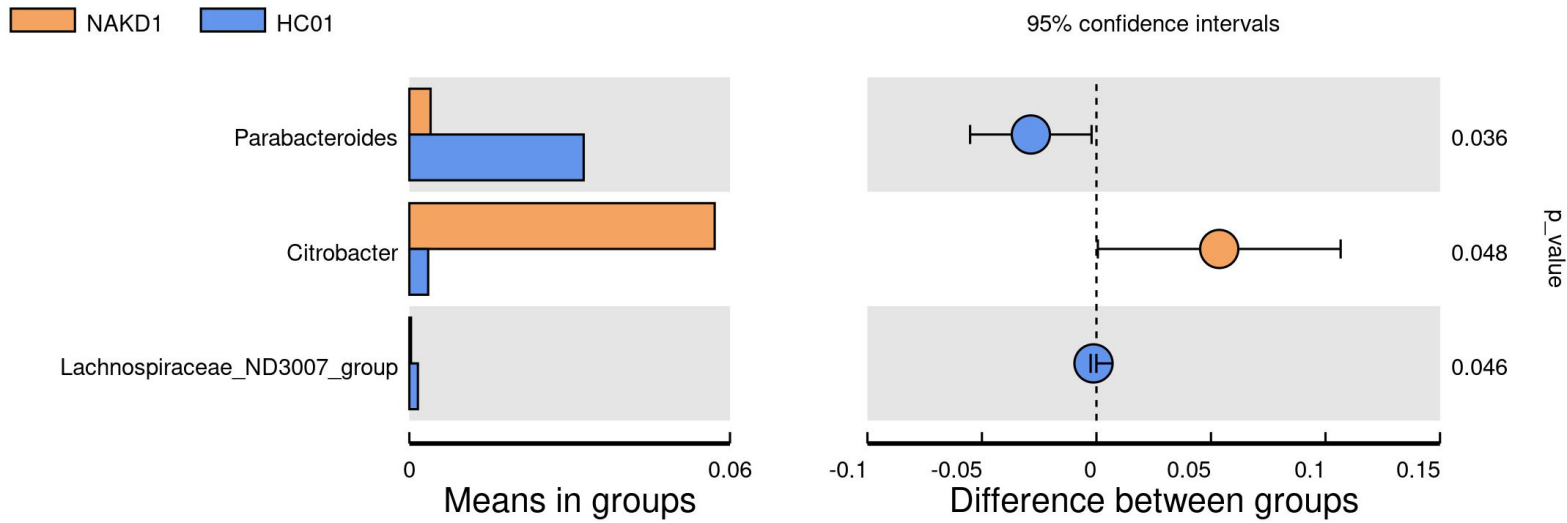
T-test at genus level between NAKD1 and HC01.

There was no statistically significant difference in the age composition ratio between the NAKD1 and HC01 groups. 613 OTUs were obtained from the fecal specimens of the NAKD1 group, which was a decrease compared to 661 OTUs in the HC01 group (**Supplementary Figure S11**). No statistically significant difference in the alpha diversity between the two groups (**Supplementary Figure S12**). The composition of the two groups was similar at the phylum level of categorization, with the main components of the NAKD1 group being Firmicutes (34.3%), Bacteroidota (31.0%), Proteobacteria (27.7%), and Actinobacteriota (5.5%). The NAKD1 group was characterized by a higher proportion of *Bacteroides* (29.5%) at the genus level. *Citrobacter* was up-regulated in the NAKD1 group, while *Parabacteroides* (p=0.036) and *Lachnospiraceae-ND3007-group* (p=0.046) were down-regulated (**Figure 7**).

#### 3.4.2 Potential bacterial biomarkers of NAKD1 patients

**Figure 8:**
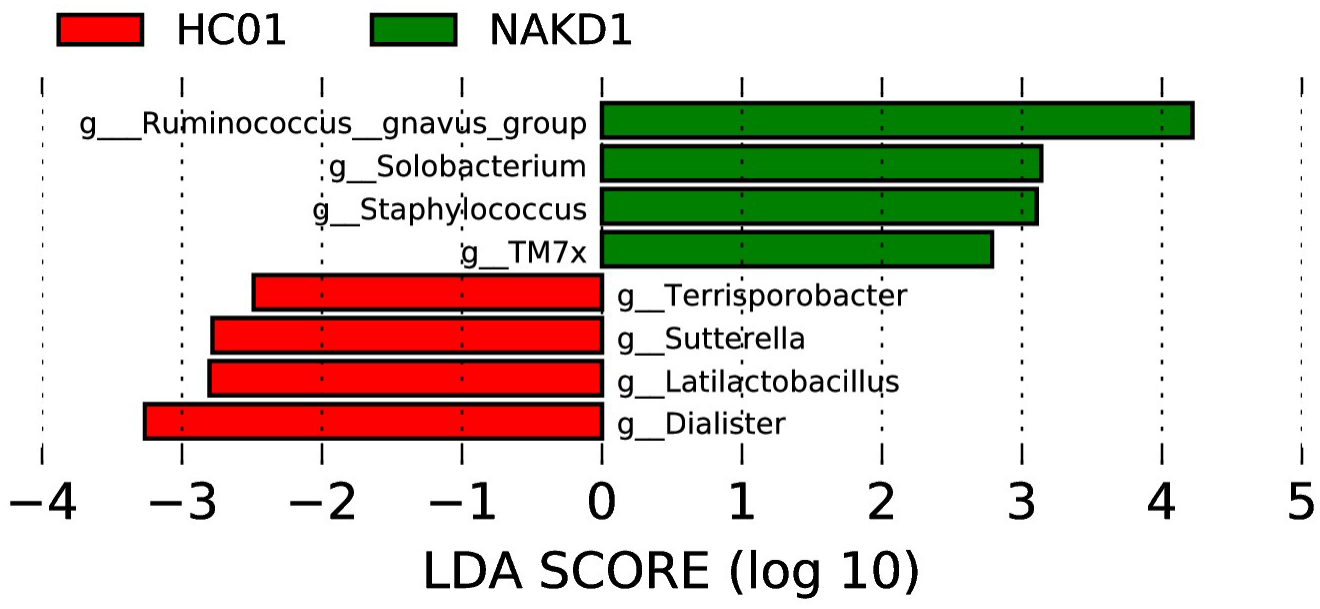
LEfSe of NAKD1 and HC01 at genus level.

The LEfSe analysis at the genus level (with an LDA score of log10 > 2.0 and p < 0.05) was conducted to identify potential bacterial biomarkers associated with NAKD1. This analysis revealed that several genera, including *Ruminococcus_gnavus_group, Solobacterium, Staphylococcus*, and *TM7x*, were significantly enriched in NAKD1 patients. Conversely, certain genera exhibited a decrease in abundance among NAKD1 patients, including *Terrisporobacter, Sutterella, Latilactobacillus*, and *Dialister* (**Figure 8**).

#### 3.4.3 Functional capability analysis

The biological function of NAKD1 patients was distinguished by PICRUSt, which revealed elevated activity in environmental information processing at the KEGG level 1 (**Supplementary Figure S13A**). Concurrently, there was a decrease in metabolism, genetic information processing, and organismal systems. At the KEGG level 2 level, the NAKD1 group demonstrated significantly higher activity in cellular processes and signaling, transcription, metabolism, cell motility, and signal transduction. Conversely, the HC01 group exhibited higher levels in amino acid metabolism, replication and repair, translation, and nucleotide metabolism (**Supplementary Figure S13B**). Furthermore, a multitude of KEGG pathways manifested statistically significant differences at level 3 (**Supplementary Figure S13C**). Of particular interest are ABC transporters, transcription factors, two-component systems, secretion systems, and bacterial motility proteins, which were found to be significantly elevated in the NAKD1 group.

#### 3.4.4 Relationship between CAL and gut microbiota changes in NAKD1 patients

The present study has revealed a lack of statistically significant correlation between CALs and gut microbiota diversity in the NAKD2 group. At the phylum level, the predominant composition of those with CALs was Bacteroidota (35.9%), Firmicutes (39.1%), Proteobacteria (19.1%), and Actinobacteriota (3.3%). The remaining subjects exhibited a composition consisting of Bacteroidota (28.5%), Firmicutes (31.9%), Proteobacteria (32.0%), and Actinobacteriota (6.6%), with no statistically significant difference observed. At the genus level, the CALs group was predominantly comprised of Bacteroides (34.1%) and Faecalibacterium (11.1%), while the other group was primarily composed of Bacteroides (27.2%), Escherichia-Shigella (9.3%), and Faecalibacterium (7.2%). Agathobacter exhibited a decrease in expression at the phylum level (p=0.048). LEfSe analysis (LDA score (log10) > 2.0, p < 0.05) revealed that Bilophila was enriched in CAL at the genus level. PICRUSt analysis indicated that there was no significant difference in the functional gene composition of gut microbiota between the two groups.

### 3.5 NAKD2 group vs HC02 group

#### 3.5.1 Differences in gut microbiota composition between NAKD2 group and HC02 group

**Figure 9:**
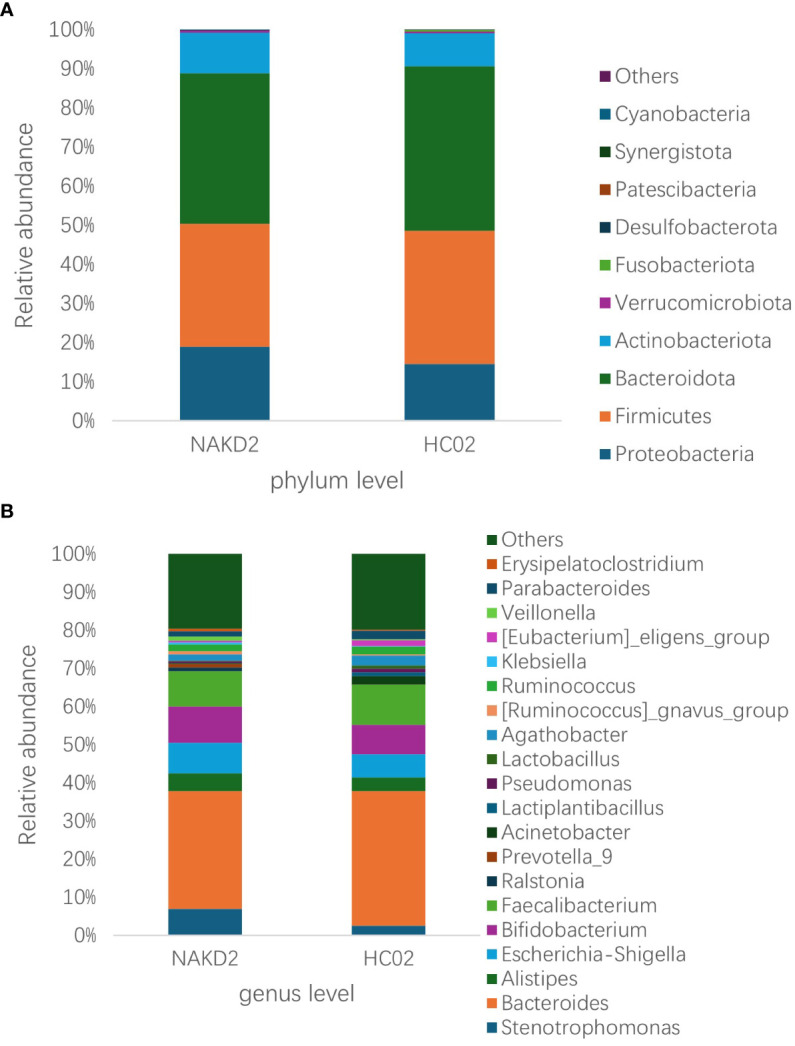
Relative abundance of species at the **(A)** phylum and **(B)** genus level for NAKD2 and HC02.

**Figure 10:**
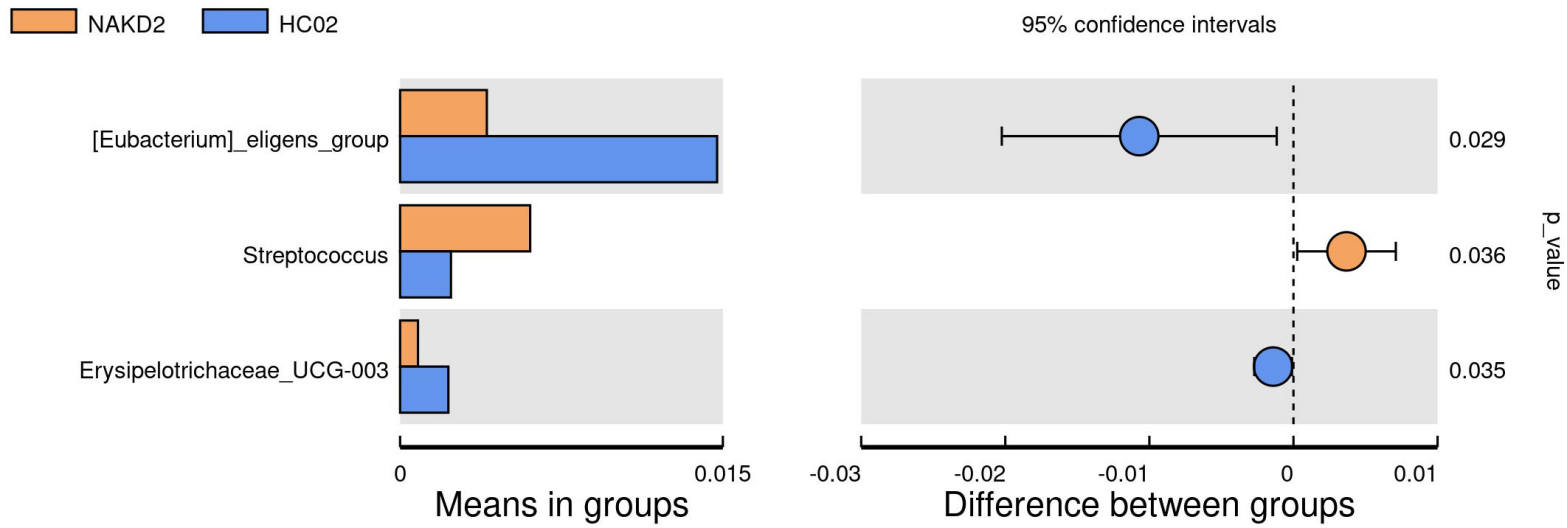
T-test at genus level between NAKD2 and HC02.

There was no statistically significant difference in the age composition ratio between the NAKD2 and HC02 groups. 893 OTUs were obtained from the fecal specimens of the NAKD2 group, which was more than the 844 OTUs obtained from the HC02 group, and the Venn diagrams showed the characteristic sequence of the groups (**Supplementary Figure S14**). There was no statistically significant difference in the alpha diversity between the two groups (**Supplementary Figure S15**). The composition of the two groups was similar at the phylum level (**Figure 9A**), and there was no statistically significant difference in the composition. NAKD 2 group was composed of Firmicutes (31.5%), Bacteroidota (38.5%), Proteobacteria (19.0%), and Actinobacteriota (10.3%). The HC02 group was composed of Firmicutes (34.0%), Bacteroidota (42.1%), Proteobacteria (14.5%), and Actinobacteriota (8.3%). At the genus level, both groups were characterized by a higher proportion of *Bacteroides* (**Figure 9B**), with *Streptococcus* up-regulated in the NAKD2 (p=0.036), while *Eubacterium-eligens-group* (p=0.029) and *Erysipelotrichaceae_UCG_003* (p=0.035) were down-regulated (**Figure 10**).

#### 3.5.2 Potential bacterial biomarkers of NAKD2 patients

**Figure 11:**
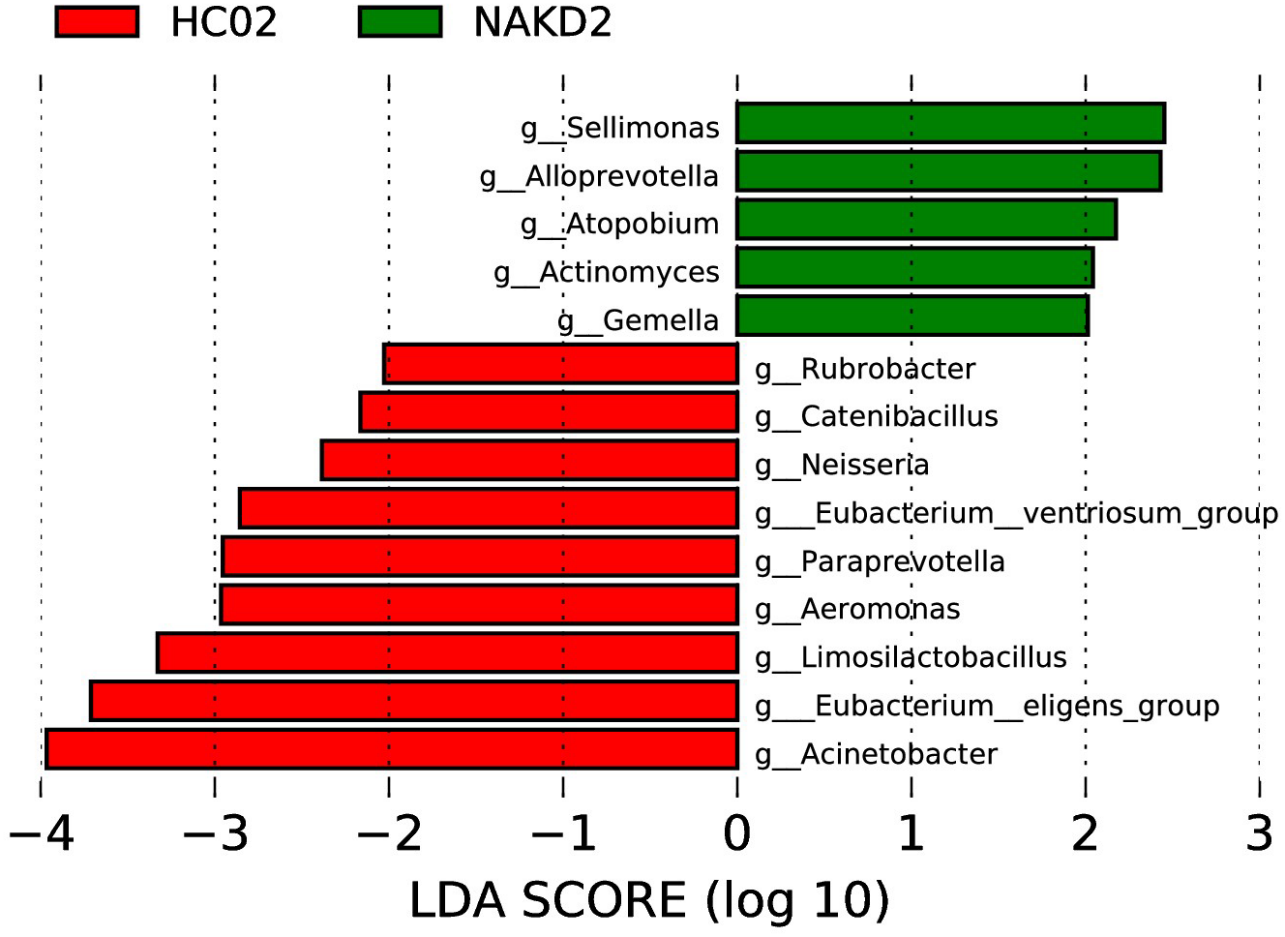
LEfSe of NAKD2 and HC02 at genus level.

LEfSe analysis at the genus level (LDA score (log10) > 2.0, p < 0.05) was performed to find the potential bacterial biomarkers associated with NAKD2. *Sellimonas, Alloprevotella, Atopobium, Actinomyces*, and *Gemella* were enriched in NAKD patients. Some are decreased in NAKD patients, including *Rubrobacter, Catenibacillus, Neisseria, Eubacterium_ventriosum_group, Paraprevotella, Aeromonas, Limosilactobacillus, Eubacterium_eligens_group*, and *Acinetobacter* (**Figure 11**).

#### 3.5.3 Functional capability analysis

PICRUSt analysis showed that there was no significant difference between the two groups.

#### 3.5.4 Relationship between CAL and gut microbiota changes in NAKD2 patients

**Figure 12:**
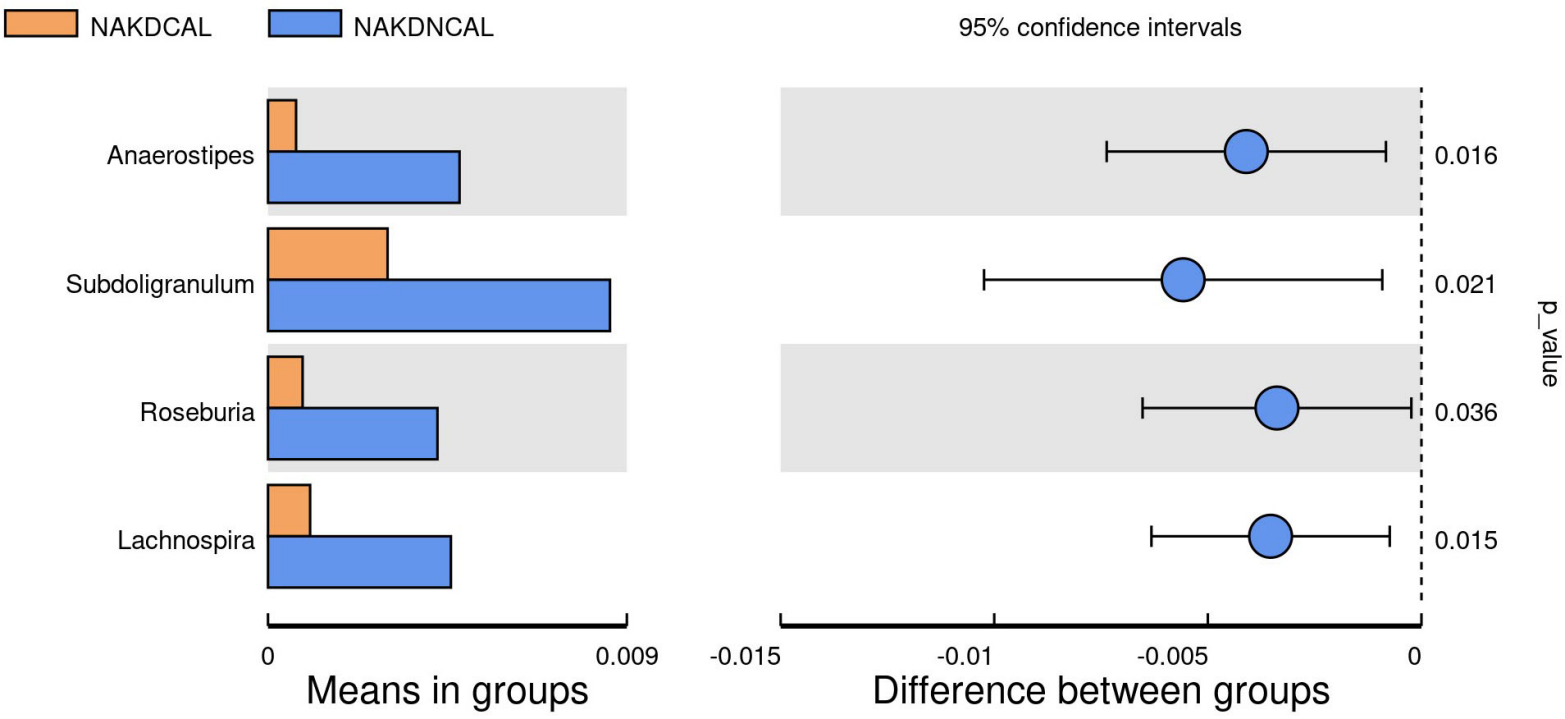
T-test at genus level between NAKDCAL and NAKDNCAL.

To explore the association between the presence or absence of comorbid CAL and gut microbiota changes during the non-acute phase of KD (duration >3 months), we performed statistical analysis between the NAKDCAL group and the NAKDNCAL group. 767 OTUs were obtained from fecal samples in the NAKDCAL group, which was a decrease compared to 594 OTUs in the NAKDNCAL group (**Supplementary Figure S16**), but the alpha diversity of the two groups was not statistically significant (**Supplementary Figure S17**). At the phylum level (**Supplementary Figure S18A**), the NAKDCAL group was mainly composed of Bacteroidota (49.1%), Firmicutes (28.7%), Proteobacteria (10.4%), and Actinobacteriota (10.8%). The AKDNCAL group consisted of Bacteroidota (34.5%), Firmicutes (32.5%), Proteobacteria (22.2%), and Actinobacteriota (10.1%). Bacteroidota was upregulated at the phylum level (p=0.038). At the genus level, the NAKDCAL group was mainly composed of *Bacteroides* (35.5%), *Alistipes* (11.3%), and *Bifidobacterium* (9.6%), whereas the NAKDNCAL group was mainly composed of *Bacteroides* (29.5%), and *Bifidobacterium* (9.6%). *Bacteroides* (29.0%), *Faecalibacterium* (10.3%), *Bifidobacterium* (9.5%), and *Stenotrophomonas* (9.5%) (**Supplementary Figure S18B**). A t-test examining all the different strains between the two groups at the genus level (**Figure 12**) showed that four strains in the NAKDCAL group showed statistically significant reductions compared to NAKDNCAL, namely *Anaerostipes* (p=0.016), *Subdoligranulum* (p=0.021), *Roseburia* (p=0.036), and *Lachnospira* (p=0.015).

**Figure 13:**
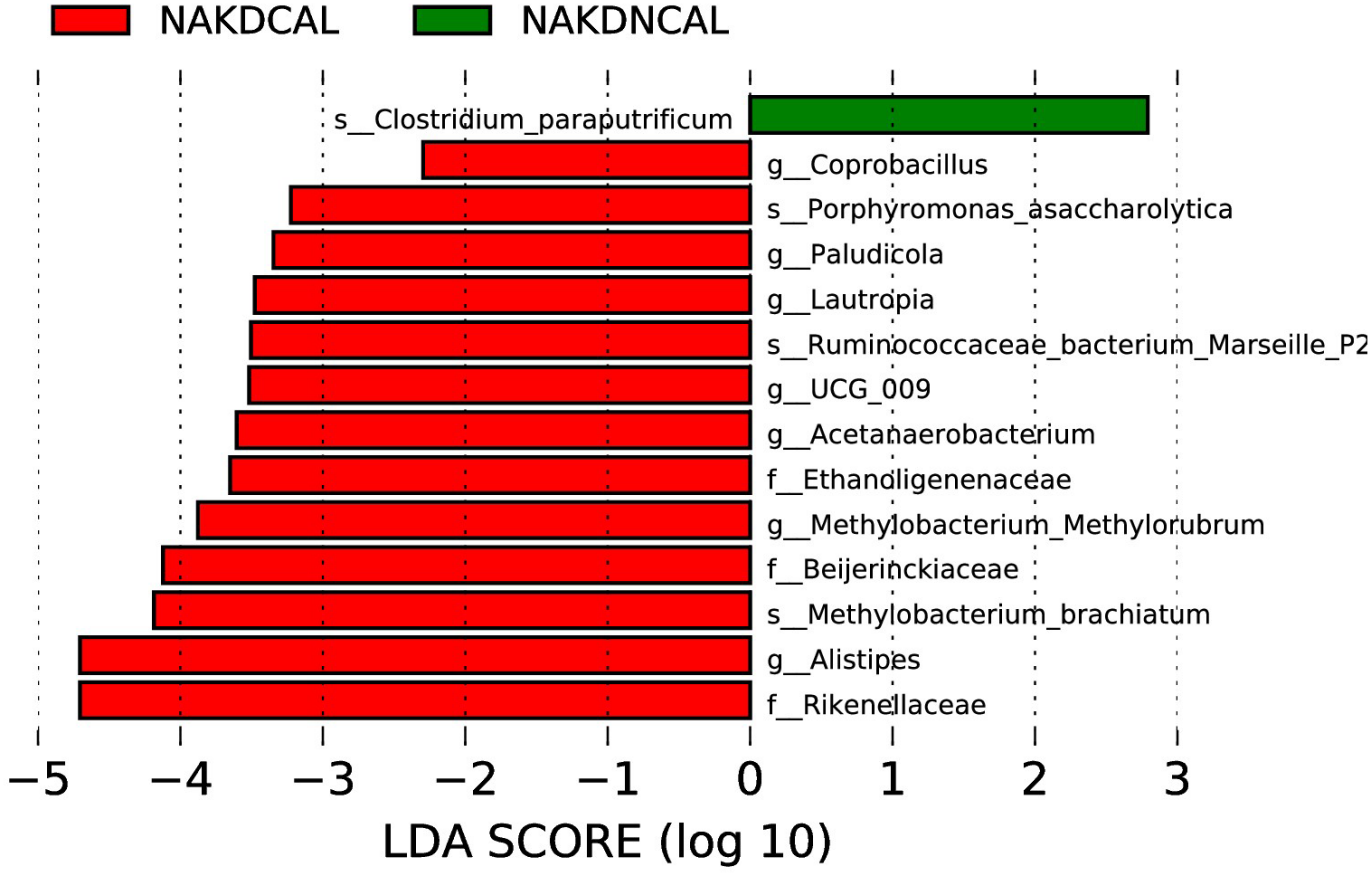
LEfSe of NAKDCAL and NAKDNCAL.

LEfSe analysis (LDA score (log10) > 2.0, p < 0.05) showed that *Coprobacillus, Paludicola, Lautropia, UCG-009, Acetanaerobacterium, Methylobacterium-Methylorubrum*, and *Alistipes* were enriched in NAKDCAL at the genus level (**Figure 13**).

We use PICRUSt to differentiate the normal biological function between the two. There was no significant difference at KEGG level 1. At KEGG level 2 (**Supplementary Figure S19A**), transport and catabolism were significantly higher in the NAKDCAL group (*P* < 0.05), while cell motility and environmental adaptation were lower (*P* < 0.05). Multiple differences in KEGG pathways were statistically significant at level 3 (**Supplementary Figure S19B**). General function prediction only, Amino sugar nucleotide sugar metabolism, Carbon fixation pathways in prokaryotes, Energy metabolism, Restriction enzyme, Lysosome, and Glycosaminoglycan degradation were higher in the NAKDCAL group (*P* < 0.05). It is worth noting that Bacterial motility proteins, Bacterial chemotaxis, and Flagellar assembly were significantly higher in the AKDNCAL group (*P* < 0.05).

## 4 Discussion

Based on our study of the role of gut microbiota in pediatric KD, we demonstrated the presence of gut microbiota dysbiosis in children with KD, particularly the downregulation of SCFAs-producing gut microbiota and the over-proliferation of conditionally pathogenic bacteria. We further investigated and confirmed the correlation between CALs and gut microbiota alterations in children with KD. Our findings may support the hypothesis that the gut microbiota may regulate energy metabolism, strengthen the intestinal barrier, exert anti-inflammatory effects by modulating the production of SCFAs, and act as a key regulator of immune cell function, thereby modulating the development of KD and CAL in children. These findings provide new insights into the role of the gut-vascular axis in the pathogenesis of pediatric KD and improve prognosis.

Akiko Kinumaki assessed longitudinal changes in gut microbiota in KD patients and reported for the first time that there was a significant difference in the composition of gut flora between acute and non-acute phases in children with KD based on metagenomic analyses of bacterial species categorization (Kinumaki et al., 2015). Jie Shen and Yoshiki Teramoto proposed that dysbiosis of gut microbiota occurs in children with acute KD and may be related to the etiology or pathogenesis of KD (Shen et al., 2020; Teramoto et al., 2023). Jie Chen’s study suggested that alterations in the gut flora of children with KD are closely related to systemic inflammation (Chen et al., 2020). Imran Khan’s study found that the dominant bacterial pathogens in the gut of KD patients were suppressed or absent after immunoglobulin/antibiotic treatment, and beneficial bacteria were colonized after treatment (Khan et al., 2020). It is easy to see that gut microbiota dysbiosis plays an integral and important role in the development of pediatric KD. In this study, we analyzed the intergroup variability and functional prediction of intestinal flora in AKD, NAKD1, NAKD2, and the corresponding control groups, respectively, to further investigate and explore their effects on CAL. We did not perform a longitudinal comparative analysis of the gut microbiota of children with KD because the specimens were not obtained from the same child at each of the three stages of the disease.

The patients with AKD were hospitalized and were treated with low-dose aspirin according to the guidelines following the initiation of a standardized treatment regimen. We observed significant differences in the diversity and composition of the gut flora between the AKD group and healthy controls. Alpha diversity analysis revealed significant differences in microbial abundance and diversity, suggesting that the KD gut microbiota is reduced in abundance and diversity. In the AKD group, Proteobacteria increased and Bacteroidota decreased at the phylum level. At the genus level, *Enterococcus* and *Escherichia-Shigella* increased, while *Bacteroides, Faecalibacteriu*, and *Agathobacter* were significantly decreased in the AKD group. LEfSe analysis showed that Enterococcus, *Alloprevotella, Blautia, Romboutsia, Senegalimassilia, Burkholderia-Caballeronia-Paraburkholderia, Actinomyces* might be the potential bacterial biomarkers in children with the acute phase of KD. This has some similarities and some differences with previous studies. Children in the acute phase of KD have gut microbiota dysbiosis. However, the differential strains are not entirely consistent, which may be related to several factors. Firstly, the prevalence of KD is notable among children in the younger age group. During this period, the intestinal tract exhibits early developmental characteristics of an unstable community structure and low flora maturity (Xiao et al., 2021). This may be a contributing factor to the characteristic structural instability of the intestinal flora in children with AKD. Secondly, an individual’s genetic predisposition may determine the response of children with AKD to environmental factors and the composition of the intestinal flora. Certain genetic susceptibilities may render an individual more susceptible to intestinal dysbiosis during infection or inflammation. Thirdly, environmental factors, such as geography, diet, and hygiene, have been demonstrated to exert a substantial influence on the composition of gut flora. Consequently, fluctuations in gut flora among children with AKD residing in disparate regions are not entirely congruent. Fourthly, the enhancement of living standards has resulted in the increased accessibility of antibiotics. In the presence of febrile symptoms, parents often misconstrue them as manifestations of certain infectious diseases and, on occasion, administer antibiotics to their children based on their personal experience. This practice may culminate in a diminution of beneficial gut bacteria. Finally, the degree of elevation in infection indicators and inflammatory factor levels in children with AKD varies across studies, highlighting the need for individualized assessments. Variations in inflammatory states may compromise intestinal barrier function, consequently altering the composition of intestinal flora. The pathogenesis of KD is unclear and is often thought to be related to the activation of the immune system in genetically susceptible children caused by a specific infection, and the abnormal response of the immune system may be related to the diversity and composition of the intestinal flora. The gut flora of children with AKD had a downregulated microbiota producing SCFAs and an overrepresentation of conditionally pathogenic bacteria compared to controls. PICRUSt was used for functional gene composition of the intestinal flora of AKD. Membrane transport, metabolism, xenobiotic biodegradation and metabolism, signal transduction, and infectious diseases were significantly higher in the AKD group, while amino acid metabolism, replication, and repair, metabolism of cofactors and vitamins, enzyme families, biosynthesis of other secondary metabolites, cell growth, and death, the endocrine system, environmental adaptation, the nervous system, and metabolic diseases were decreased. Notably, the two-component system, secretion system, and phosphotransferase system (PTS) were significantly increased in the AKD. SCFAs like acetate, propionate, and butyrate are important bacterial fermentation metabolites regulating many important aspects of human physiology.

SCFAs produced by intestinal bacteria may promote disease states by activating the immune system through defective intestinal barriers, suppressing pro-inflammatory cytokines, and compromising vascular integrity, causing systemic inflammatory responses (Dicks, 2024). Butyrate maintains intestinal barrier integrity by promoting epithelial tight junctions, serving as a fuel for ATP production in colonocytes, and modulating the immune system (Salvi and Cowles, 2021; Hays et al., 2024). The two-component system regulates ABC transporter proteins, immunity, and resistance in the production of antimicrobial peptides (Ahmad et al., 2020). The bacterial type VI secretion system (T6SS) plays a crucial role in the adaptation of pathogenic bacteria, often through the delivery of virulence effectors to target cells (Jiang et al., 2024). Xiaoli Jiang’s research has revealed that the host gut microbiota can be remodeled through the intricate interplay between T6SS-mediated bacterial competition and altered host immune responses (Jiang et al., 2024). Wanli Zhou concluded that both IIC and IID components of the mannose PTS play an important role in the specific recognition between the bacteriocin-receptor complex and the immunity protein PedB (Zhou et al., 2016).

Regarding whether CAL in the AKD affects the species diversity, abundance, and compositional structure of the intestinal flora, our study suggests that the answer is negative. However, LEfSe analysis showed that the *Burkholderia-Caballeronia-Paraburkholderia, Hungatella*, and *Clostridium-innocuum group* was up-regulated in the presence of coronary damage. The *Burkholderia-Caballeronia- Paraburkholderia* is a central taxon within the nasopharyngeal microbial network in type 2 severe asthma, which is ubiquitous throughout the airways and is involved in bacterial functions, including inflammatory and steroidogenic responses (Zhang et al., 2025). In addition, it has been shown that its relative abundance is reduced during adalimumab treatment in patients with ulcerative colitis (Oh et al., 2024). *Hungatella* is usually considered a non-pathogenic component of the human gut microbiome, but its importance has gained prominence in recent studies. Some studies have proposed elevated levels of *Hungatella* in the gastrointestinal tract of patients after successful treatment with COVID-19 (Cai et al., 2024). The abundance of *Hungatella hathewayi* in tissues operated on for colorectal cancer tumors was approximately five times higher than in cancer-free tissues (Huang et al., 2023). The *Clostridium-innocuum group* is part of the normal intestinal flora and is responsible for rare, intrinsic vancomycin-resistant opportunistic infections in immunocompromised patients. There have been several recent reports suggesting that it may be a potential emerging pathogen (Cherny et al., 2021). Taken together, this information leads us to hypothesize that there is an interactive effect of CAL and gut microbiota that is not manifested in significant alterations in species diversity, abundance, and compositional structure of the gut flora in patients with acute-phase KD, but rather in microbial biological function.

NAKD1 patients had a KD duration of less than 3 months, and patients were taking oral low-dose aspirin with or without comorbid CAL, which may affect the composition of the intestinal flora. *Citrobacter* was up-regulated, while *Parabacteroides* and *Lachnospiraceae-ND3007- group* were down-regulated. *Citrobacter rodentium* is an extracellular intestinal mouse-specific pathogen used to mimic human pathogenic *Escherichia coli* infections and inflammatory bowel disease and exerts host-microbiota interactions in immunity, bioenergetics, and metabolism (Mullineaux-Sanders et al., 2019). *Parabacteroides* and *Lachnospiraceae* are physiologically characterized by carbohydrate metabolism and secretion of SCFAs, which play important roles in maintaining host-gut homeostasis (Vacca et al., 2020; Cui et al., 2022). NAKD2 patients were patients with KD duration of more than 3 months, and those with combined CAL these patients took oral low-dose aspirin for thromboprophylaxis, and the effect of oral anticoagulants on the gastrointestinal tract should not be ignored (Chi et al., 2021). *Streptococcus* was upregulated, whereas the *Eubacterium-eligens-group* and the *Erysipelotrichaceae-UCG-003* were downregulated. CAL may be associated with *Anaerostipes, Subdoligranulum, Roseburia*, and *Lachnospira* in the intestine. It was found that *Anaerostipes caccae* and lactulose synbiotics prevented and treated food allergy in mice (Hesser et al., 2024), which provides new ideas and possibilities for the flora to treat patients with KD with CAL and to improve their prognosis. *Roseburia* intestinalis is a butyric acid-producing anaerobic bacterium with positive effects on a variety of diseases including inflammatory bowel disease, atherosclerosis, alcoholic fatty liver disease, colorectal cancer, and metabolic syndrome, making it a potential next-generation probiotic (Zhang et al., 2022). In children recovering from KD, an altered intestinal flora profile has been observed, characterized by a shift in the predominance of opportunistic pathogens, such as *Enterococci*, with a concurrent decrease in beneficial bacteria, including SCFAs-producing flora. A subsequent KEGG pathway analysis of the NAKDCAL group revealed elevated levels of general function prediction, amino sugar nucleotide sugar metabolism, carbon fixation pathways in prokaryotes, energy metabolism, restriction enzyme, lysosome, and glycosaminoglycan degradation. Conversely, bacterial motility proteins, bacterial chemotaxis, and flagellar assembly demonstrated significant decreases.

In summary, our study provides compelling evidence of intestinal dysbiosis in children with KD, indicating its potential involvement in the pathogenesis of pediatric KD. The LEfSe analysis identified several potential bacterial biomarkers associated with KD and CALs. Additionally, PICRUSt analysis revealed several significantly enriched KEGG pathways. Our findings suggest that an increase in conditionally pathogenic bacteria and a decrease in beneficial bacteria during the acute phase of KD may impair intestinal mucosal function, exacerbate inflammatory and immune responses, and affect specific signaling pathways, potentially leading to coronary artery injury. However, the limitations of this study lie in the single center design, small sample size and lack of pre disease intestinal microbiological data. Due to funding constraints, the single center design leads to a relatively single data source, which may be affected by the different diet, living habits, treatment schemes and other factors caused by local space and region, resulting in the difference of species composition of intestinal flora. The research results are difficult to explain its universality, and may draw contradictory conclusions or find new differential bacteria in other research centers. This study is a cross-sectional study. Limited by the number of funds, the choice of time points, the number of patients, and the detailed grouping, the sample size in each group is small, and the compliance of some children and their families is poor, resulting in the failure to build a longitudinal database. The resulting bias may have a greater impact on the analysis of intestinal flora composition, and different conclusions may be drawn in other research centers. The causes of intestinal microbiota imbalance also include a variety of factors. This study failed to strictly control the influencing factors such as diet, genetics and individual living environment, and failed to carry out experimental verification, so the reliability of the conclusion is difficult to be confirmed in both directions. The original intention of this study is to observe the differences of intestinal flora composition in KD patients compared with the general population in a cross-sectional manner, so as to provide direction for subsequent research. In the future, we will consider further experimental verification in the follow-up study, extend the follow-up time, and build a longitudinal database to make the study more reasonable and the conclusion more reliable.

## 5 Conclusions

The richness and diversity of the gut microbiota are reduced in pediatric KD, leading to dysbiosis, particularly characterized by decreased SCFAs-producing gut microbiota and over-proliferation of conditionally pathogenic bacteria. CALs have been observed to interact with the gut microbiota during the acute phase of KD, as evidenced by the upregulation of specific bacterial groups such as *Burkholderia-Caballeronia-Paraburkholderia, Hungatella*, and the *Clostridium-innocuum group*, which may be associated with coronary damage and altered microbial biological functions. In the non-acute phase, CALs have been linked to changes in the abundance of *Anaerostipes, Subdoligranulum, Roseburia*, and *Lachnospira*. The available evidence suggests that, alongside standardized treatment (IVIG plus aspirin therapy, etc.) during the acute phase of KD, microbiota-targeted strategies could potentially improve outcomes for patients with CALs.

## Data Availability

The raw data supporting the conclusions of this article will be made available by the authors, without undue reservation.
