# Supplementary figures and images for: Unveiling the gut-heart potential connection: microbiota’s role in kawasaki disease and coronary artery lesions
Source: Front Cell Infect Microbiol. 2025 May 29;15:1560083. doi: 10.3389/fcimb.2025.1560083 (PMC12158935; doi:10.3389/fcimb.2025.1560083)

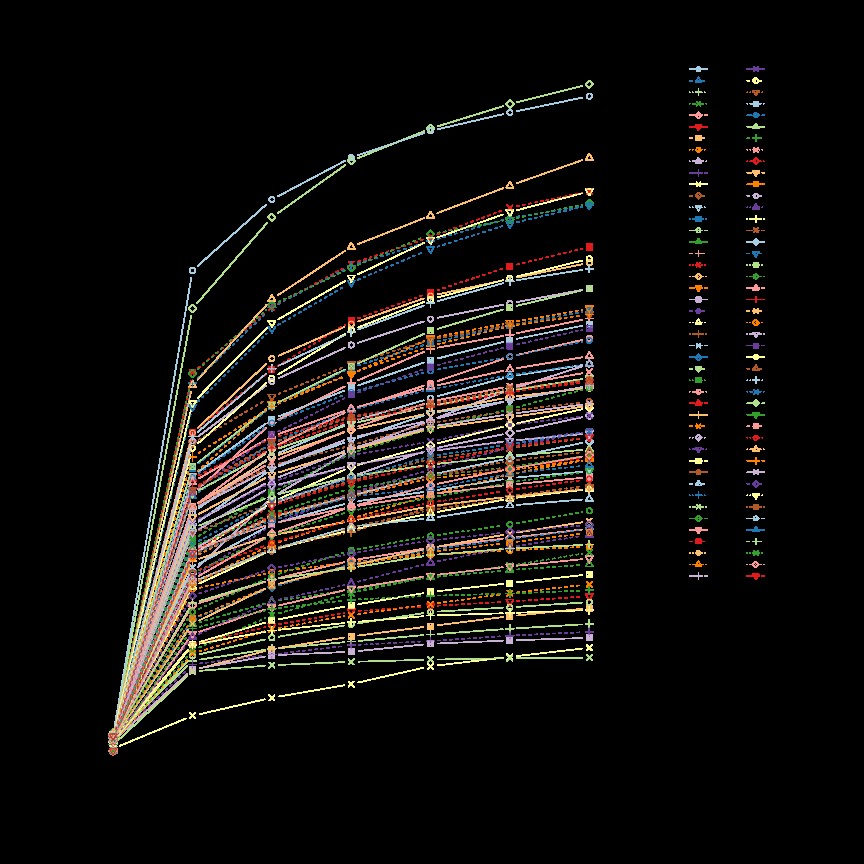

Supplement: Supplementary file 1 [file Presentation1.zip › Supplementary_Material Figures/Fig. S1 Rarefaction curve of all samples.jpg]

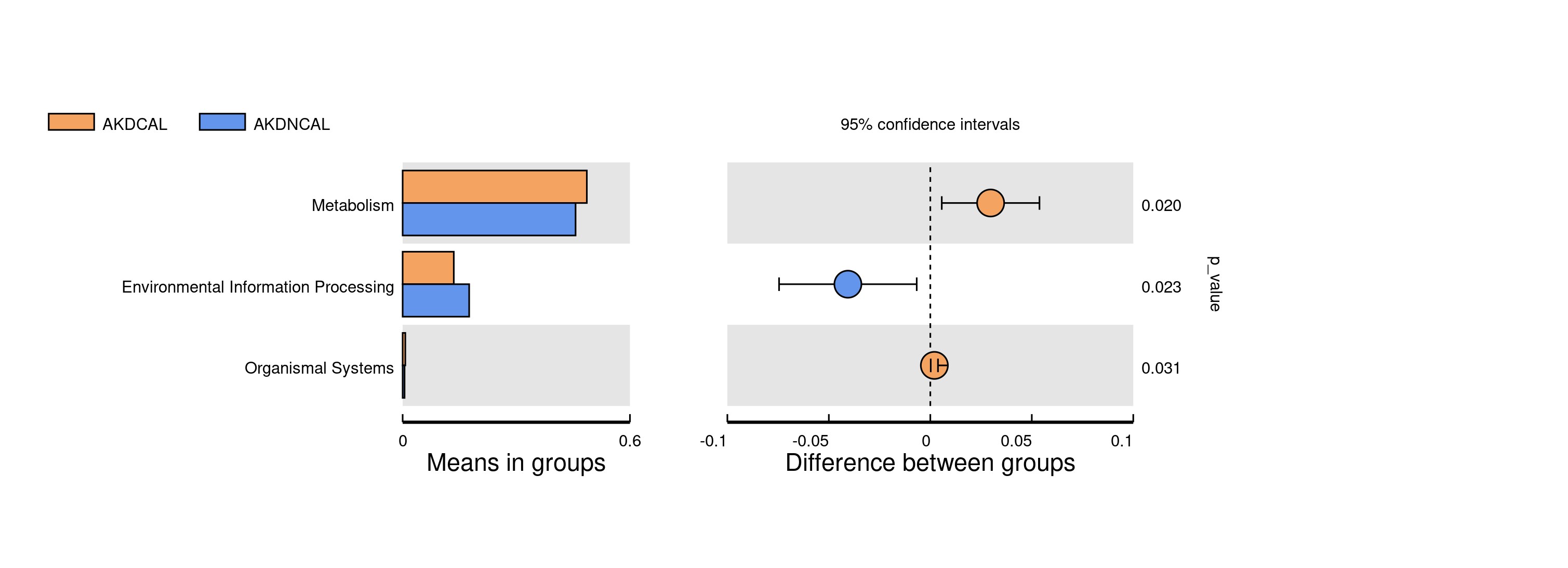

Supplement: Supplementary file 1 [file Presentation1.zip › Supplementary_Material Figures/Fig. S10A Functional capability analysis based on the KEGG pathways at level 1 between AKDCAL and AKDNCAL..jpg]

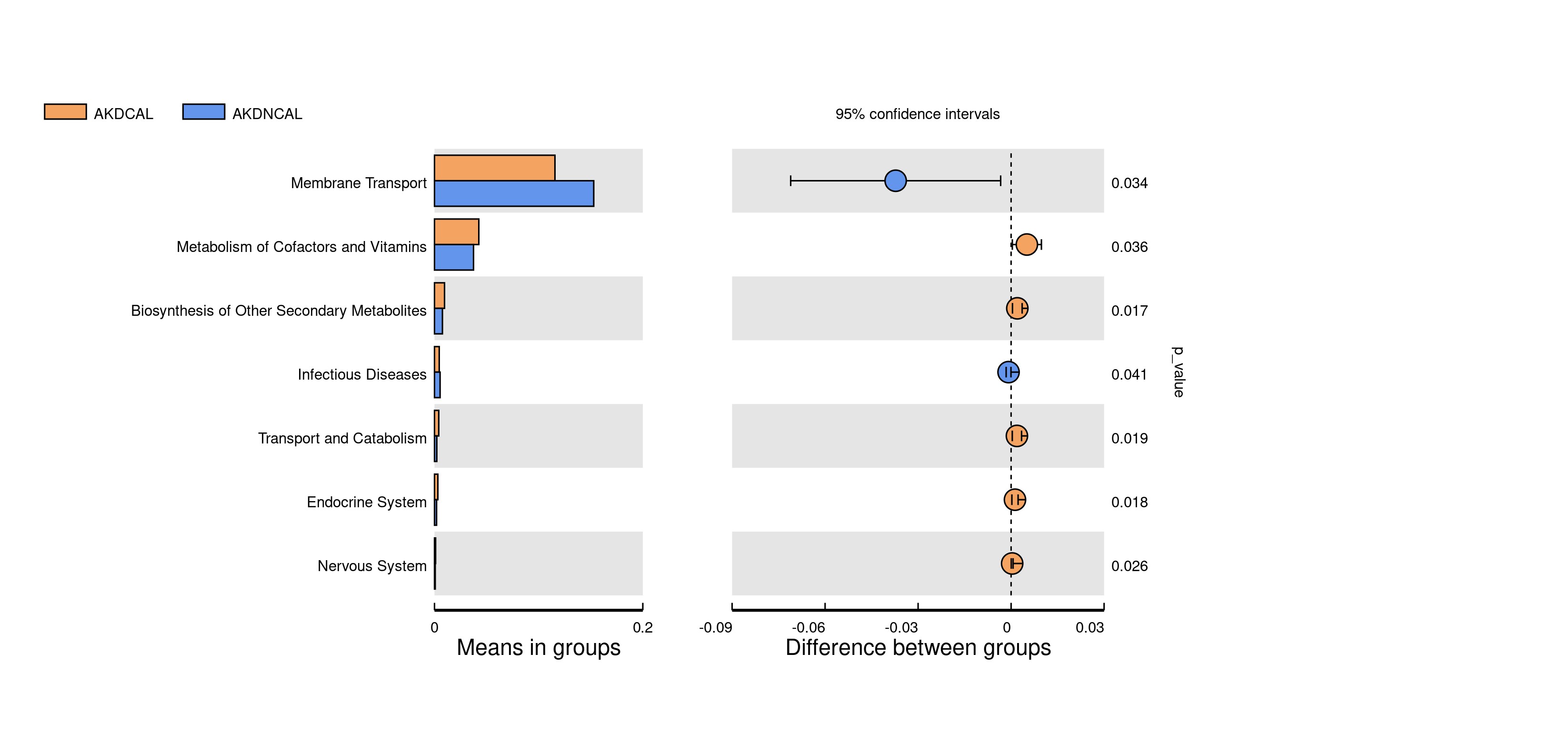

Supplement: Supplementary file 1 [file Presentation1.zip › Supplementary_Material Figures/Fig. S10B Functional capability analysis based on the KEGG pathways at level 2 between AKDCAL and AKDNCAL..jpg]

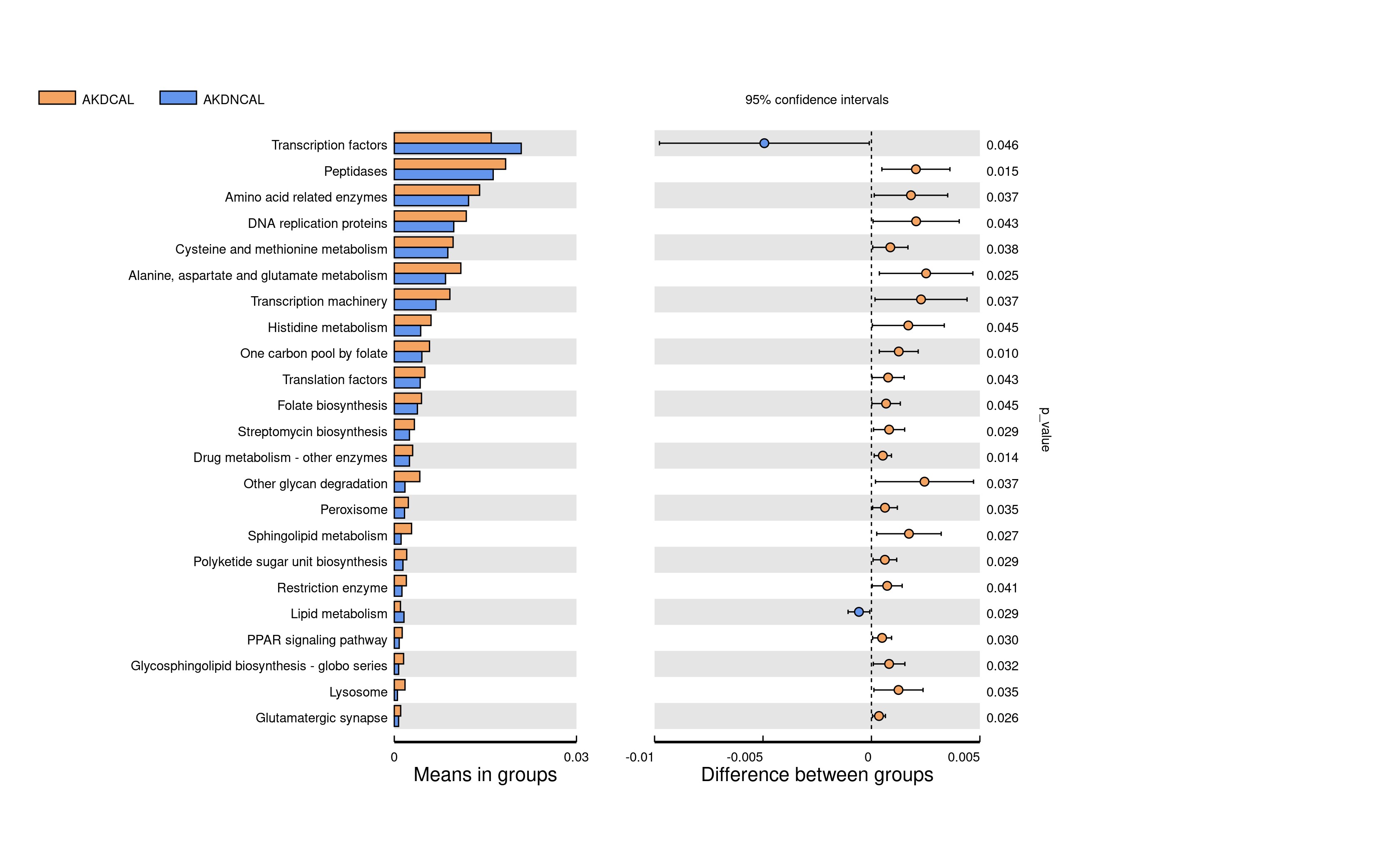

Supplement: Supplementary file 1 [file Presentation1.zip › Supplementary_Material Figures/Fig. S10C Functional capability analysis based on the KEGG pathways at level 2 between AKDCAL and AKDNCAL..jpg]

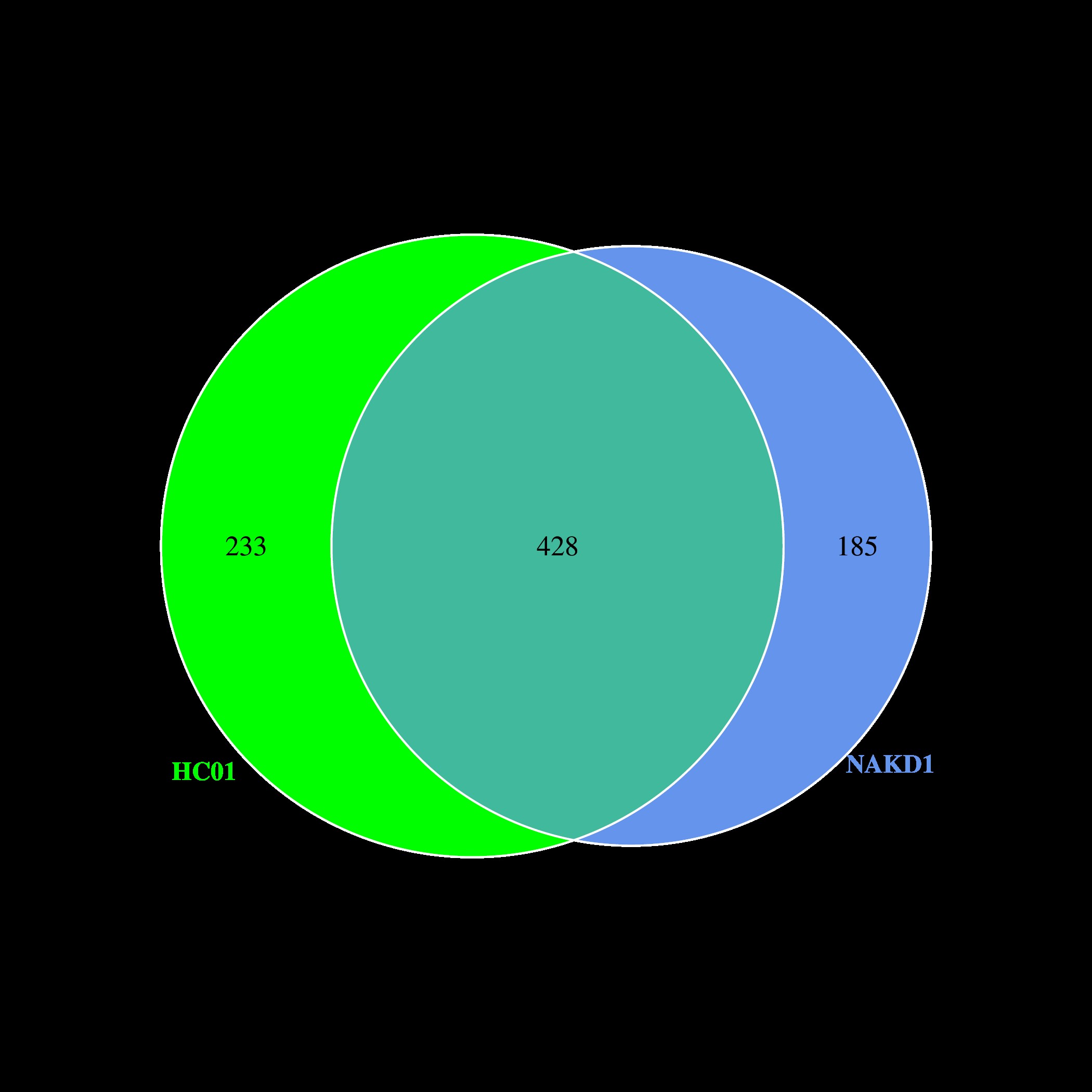

Supplement: Supplementary file 1 [file Presentation1.zip › Supplementary_Material Figures/Fig. S11 Venn diagram of NAKD1 and HC01.jpg]

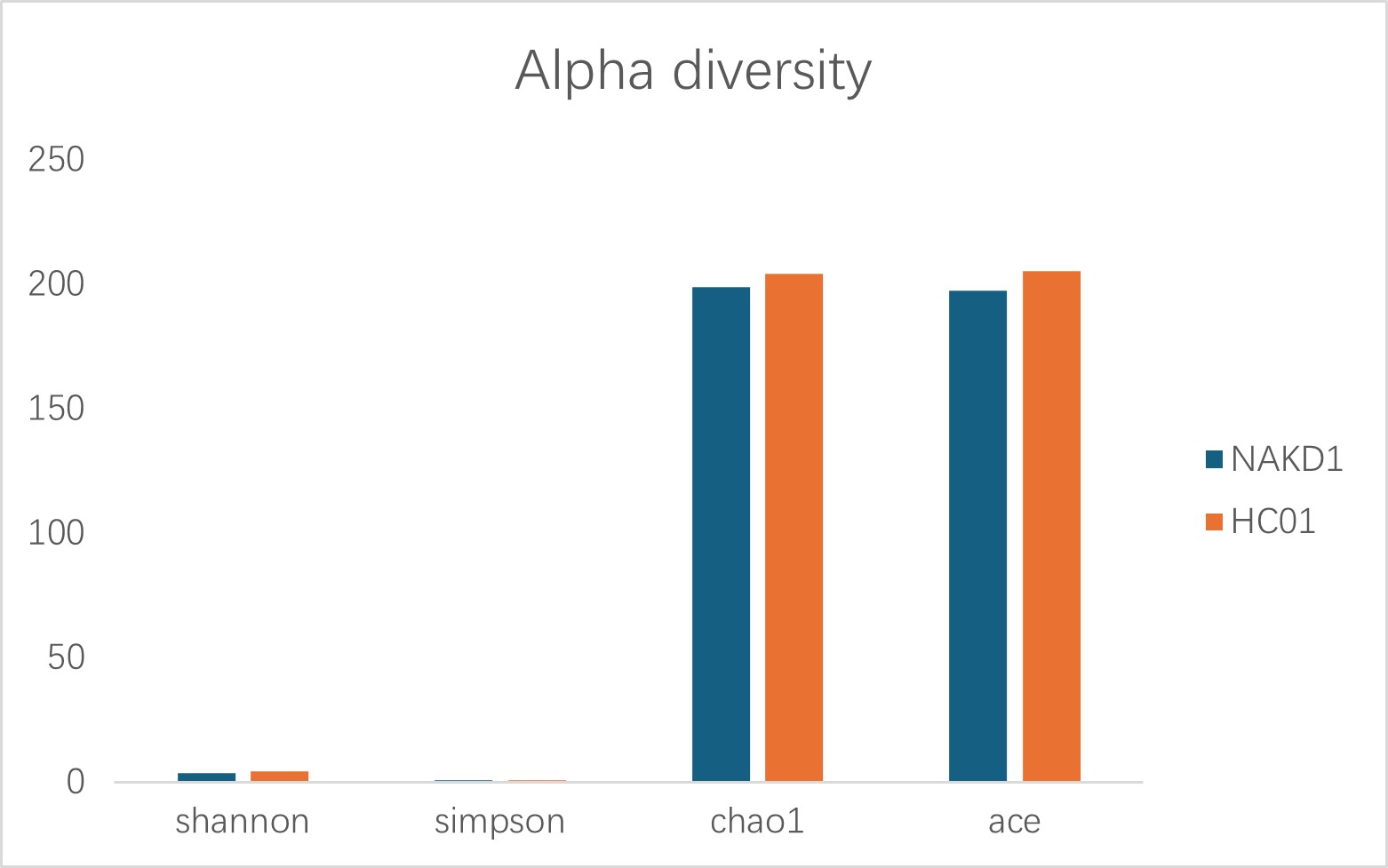

Supplement: Supplementary file 1 [file Presentation1.zip › Supplementary_Material Figures/Fig. S12 Alpha diversity index of NAKD1 and HC01.jpg]

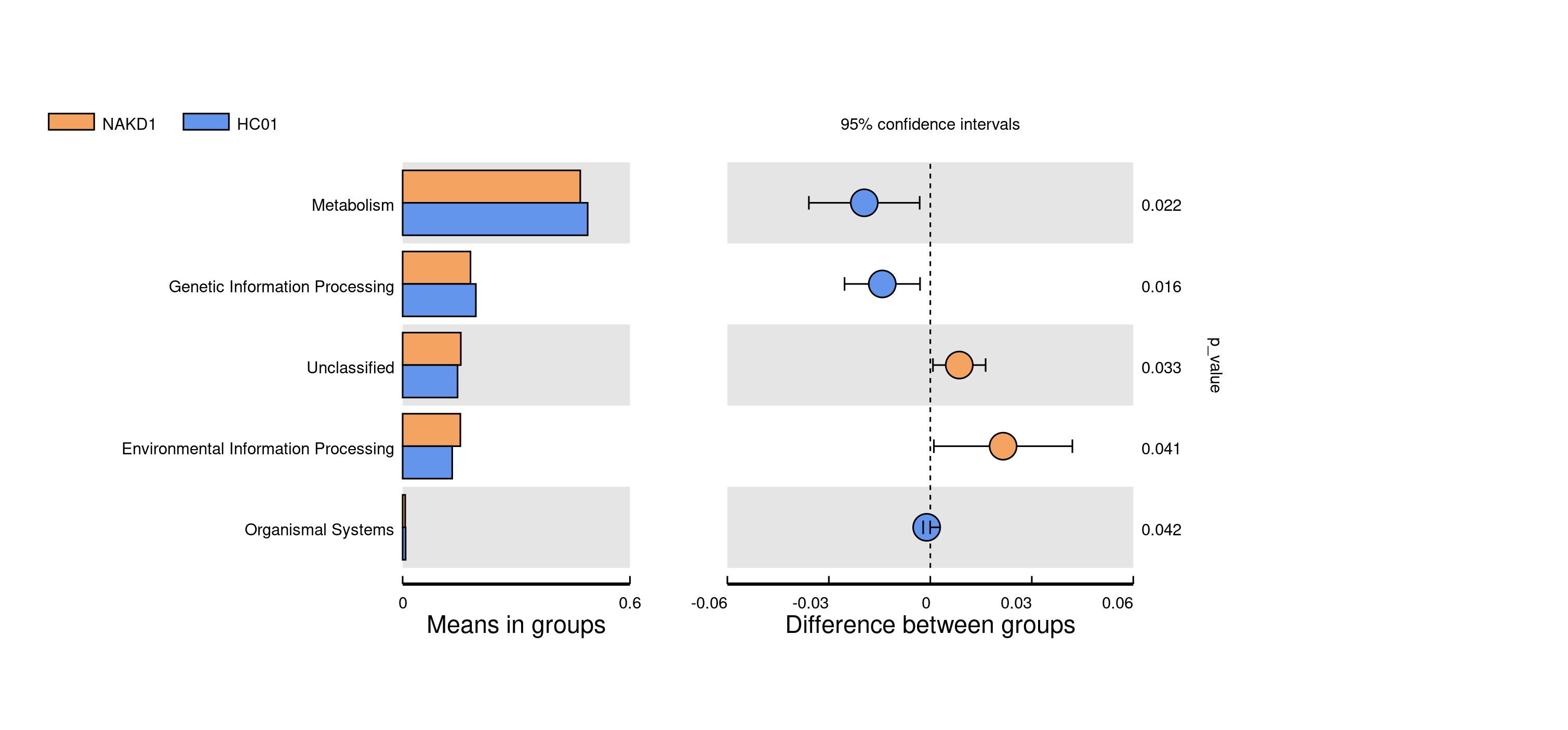

Supplement: Supplementary file 1 [file Presentation1.zip › Supplementary_Material Figures/Fig. S13A Functional capability analysis based on the KEGG pathways at level 1 between NAKD1 and HC01..jpg]

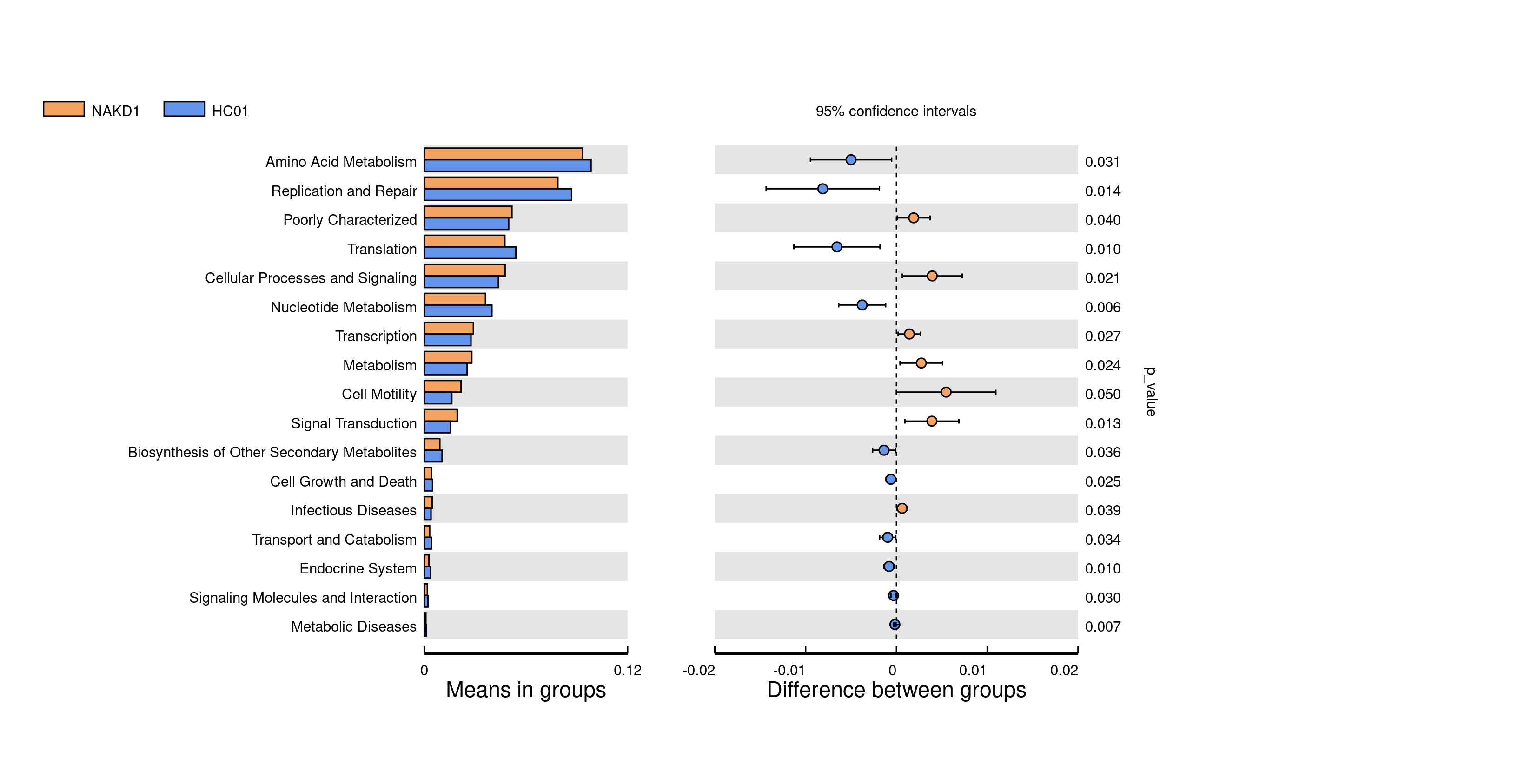

Supplement: Supplementary file 1 [file Presentation1.zip › Supplementary_Material Figures/Fig. S13B Functional capability analysis based on the KEGG pathways at level 2 between NAKD1 and HC01..jpg]

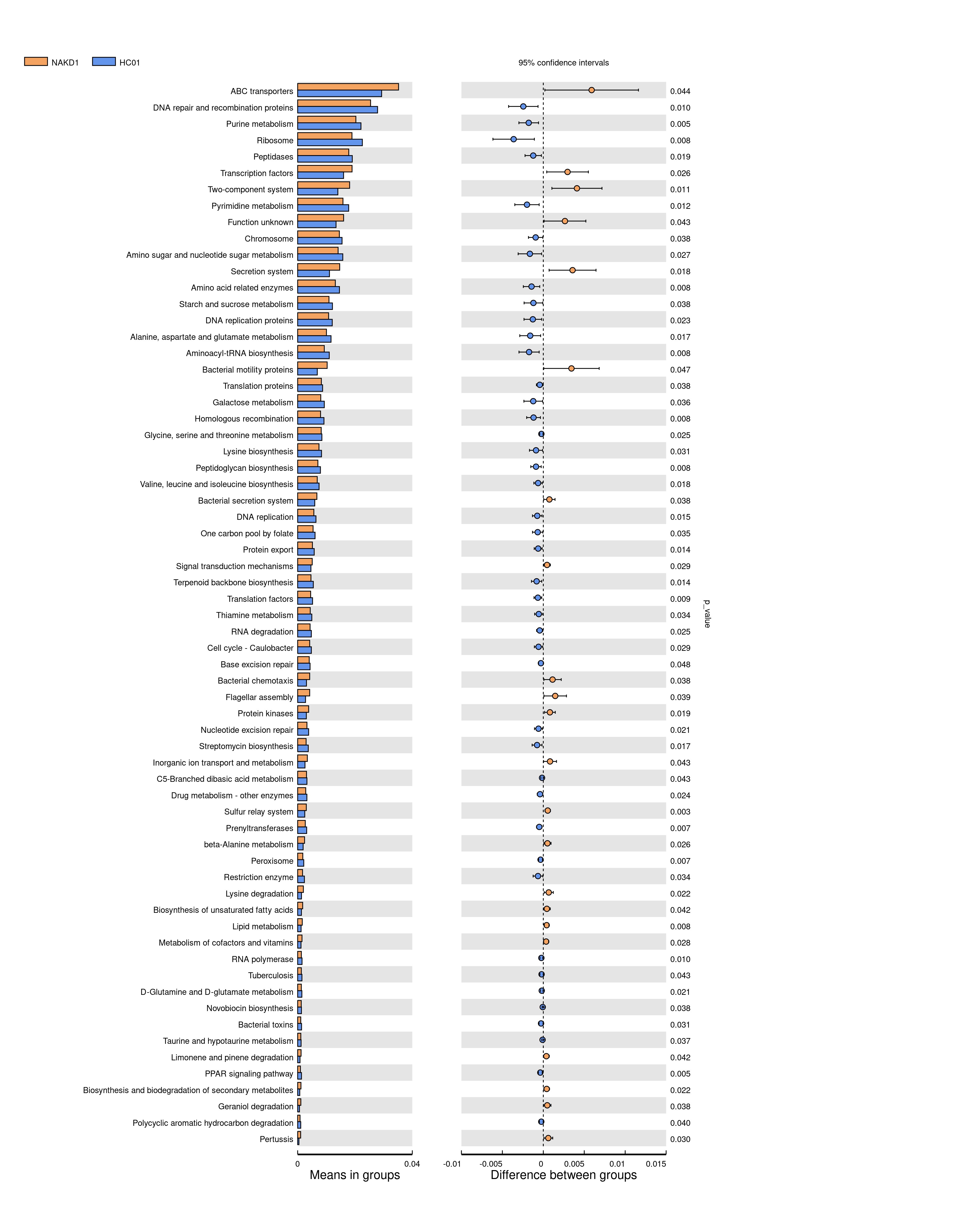

Supplement: Supplementary file 1 [file Presentation1.zip › Supplementary_Material Figures/Fig. S13C Functional capability analysis based on the KEGG pathways at level 3 between NAKD1 and HC01..jpg]

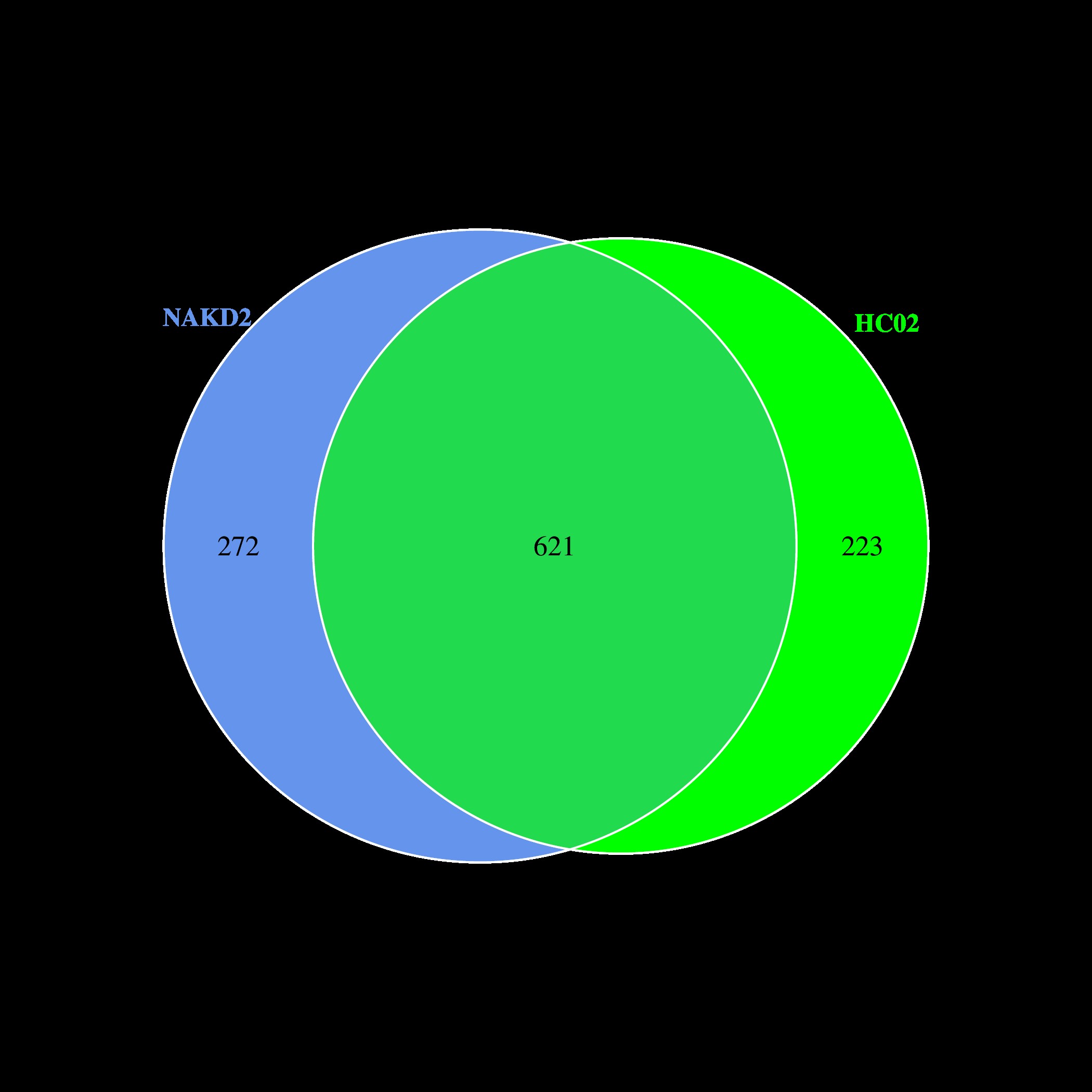

Supplement: Supplementary file 1 [file Presentation1.zip › Supplementary_Material Figures/Fig. S14 Venn diagram of NAKD2 and HC02.jpg]

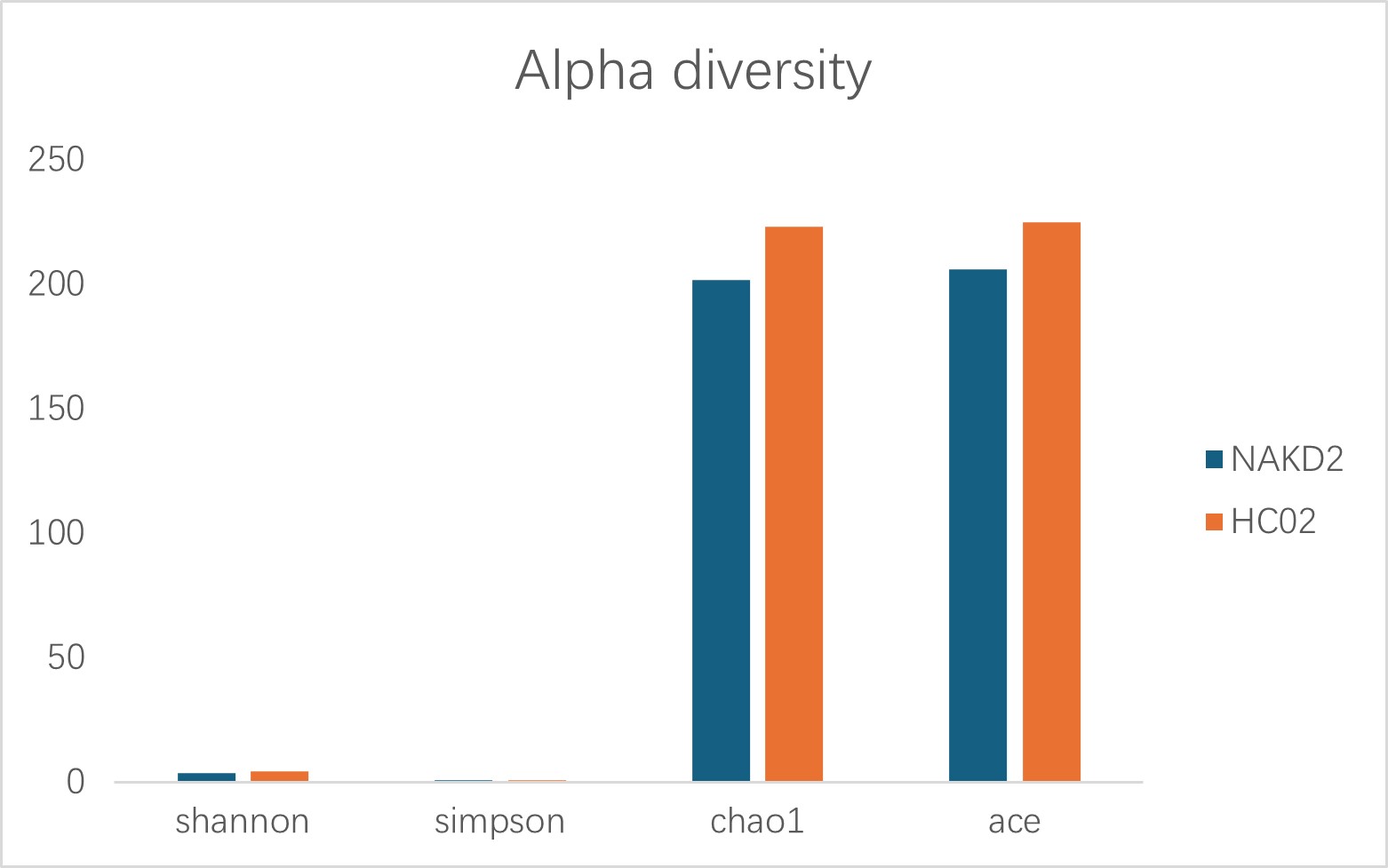

Supplement: Supplementary file 1 [file Presentation1.zip › Supplementary_Material Figures/Fig. S15 Alpha diversity index of NAKD2 and HC02.jpg]

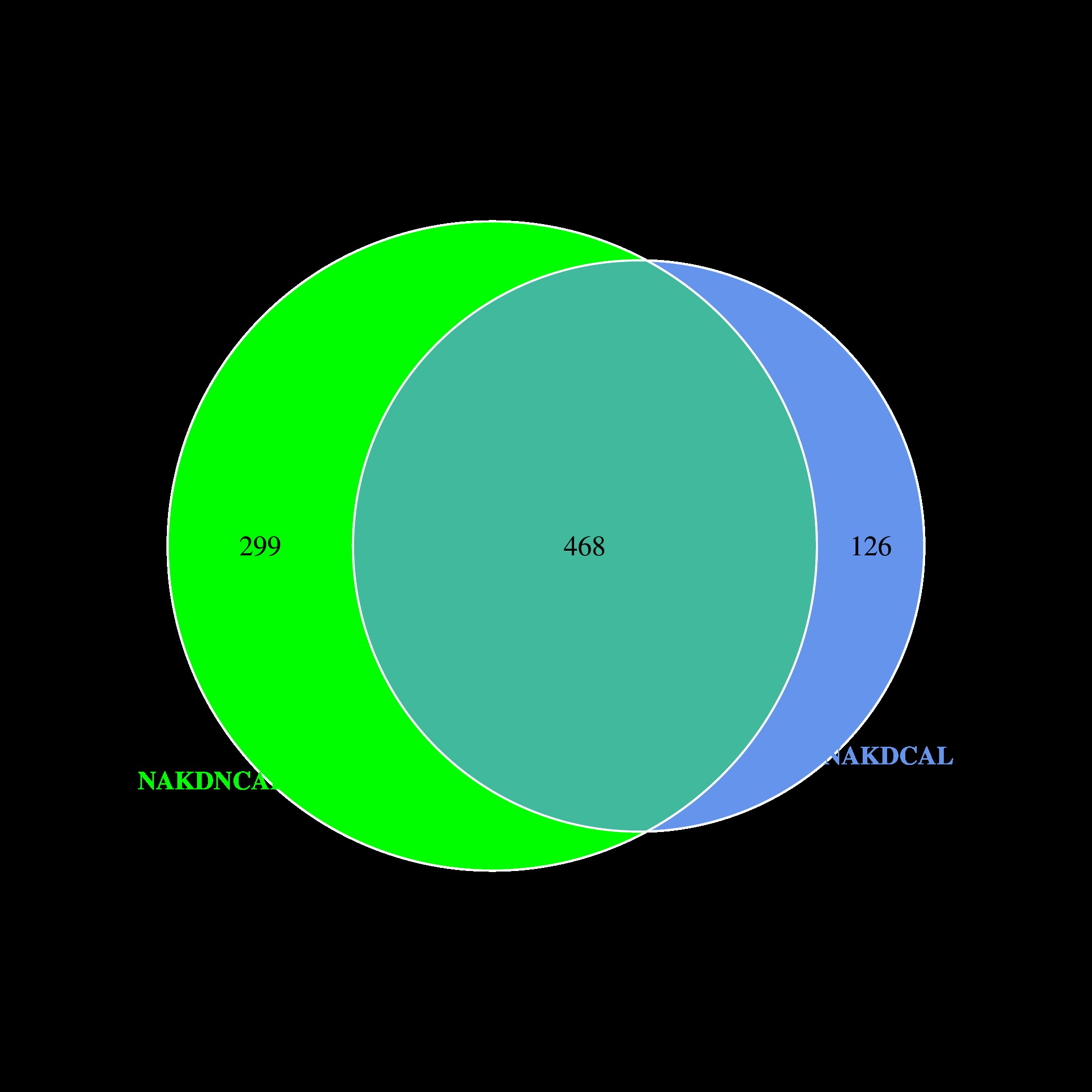

Supplement: Supplementary file 1 [file Presentation1.zip › Supplementary_Material Figures/Fig. S16 Venn diagram of NAKDCAL and NAKDNCAL.jpg]

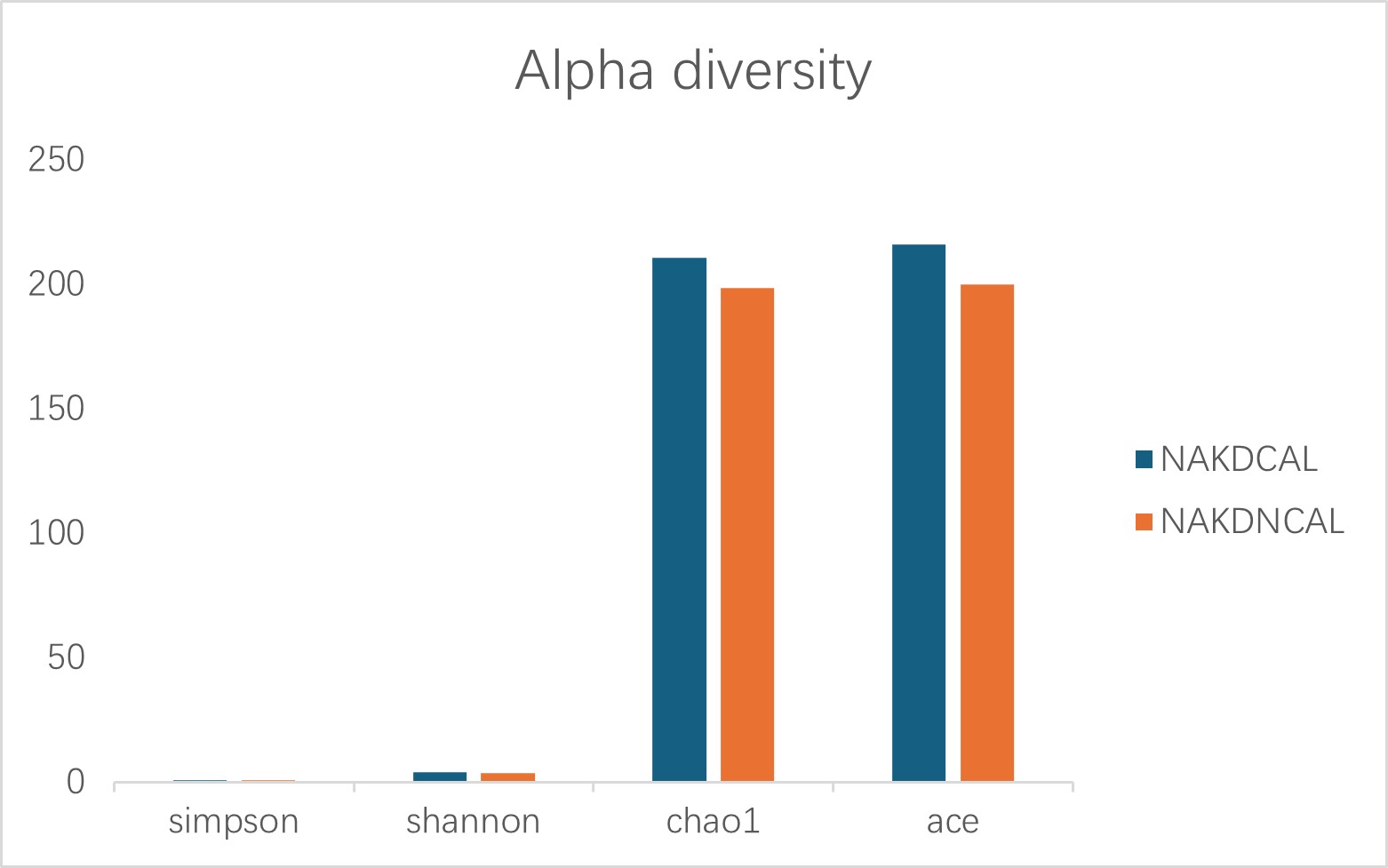

Supplement: Supplementary file 1 [file Presentation1.zip › Supplementary_Material Figures/Fig. S17 Alpha diversity index of NAKDCAL and NAKDNCAL.jpg]

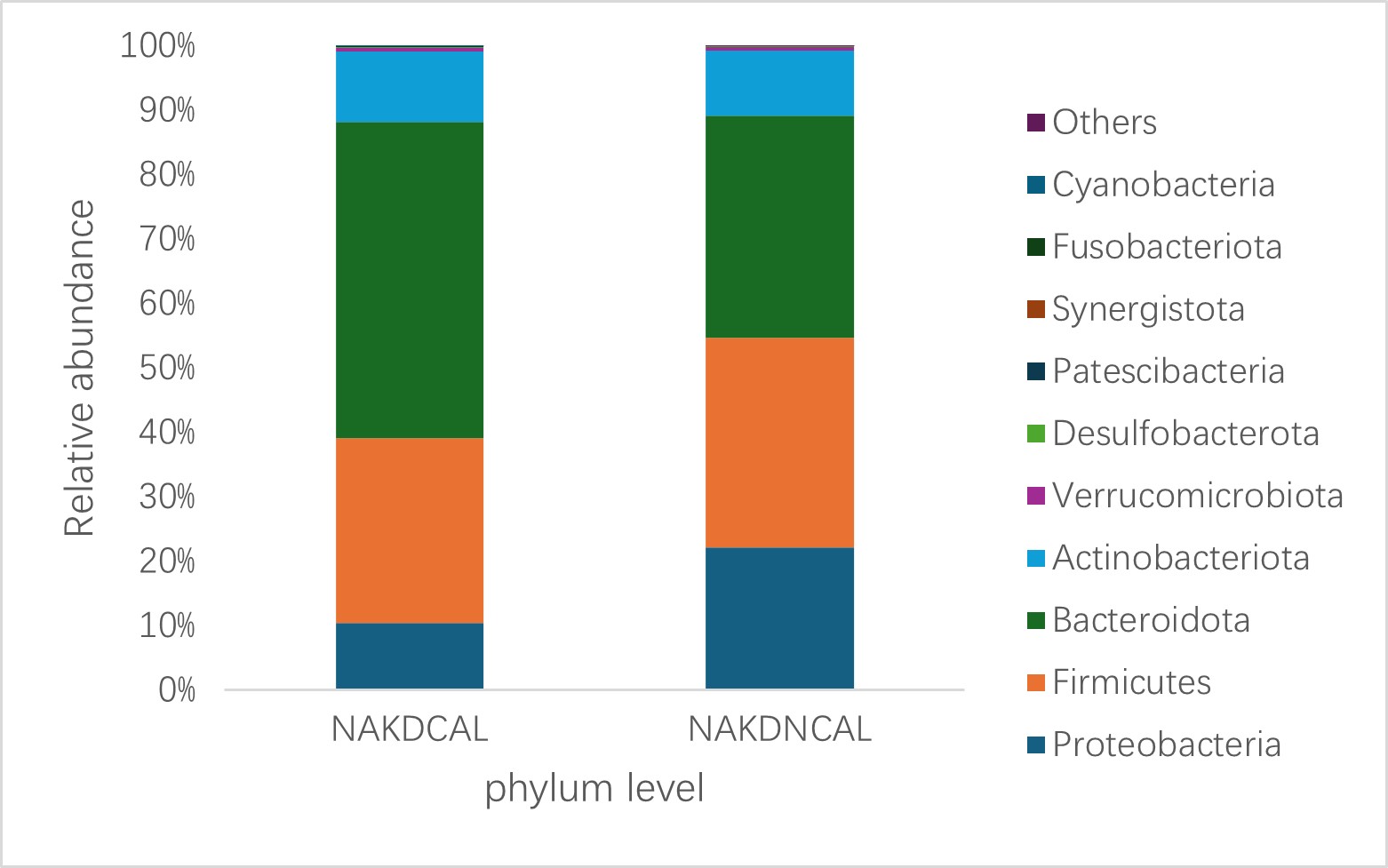

Supplement: Supplementary file 1 [file Presentation1.zip › Supplementary_Material Figures/Fig. S18A Relative abundance of species at phylum level for NAKDCAL and NAKDNCAL.jpg]

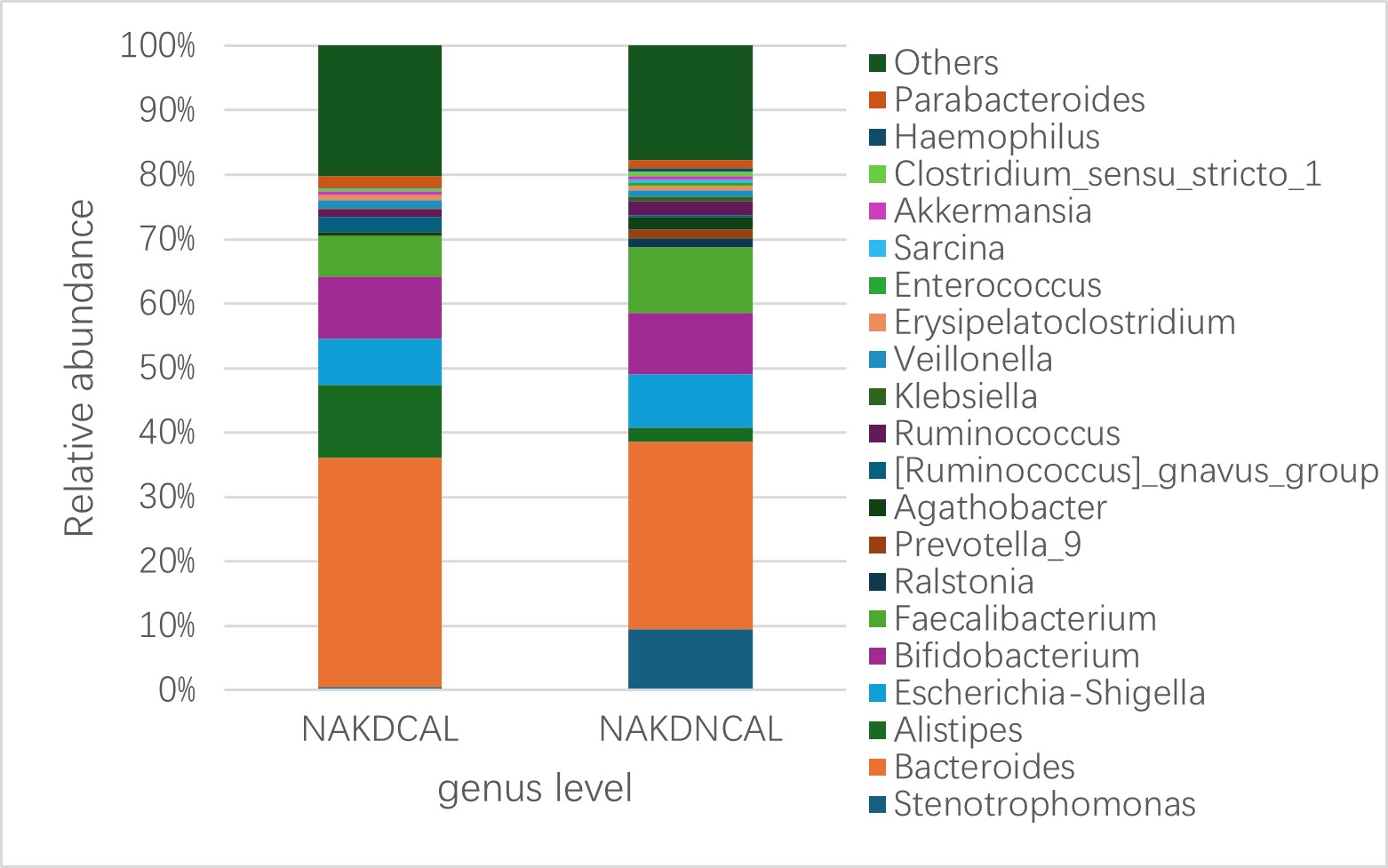

Supplement: Supplementary file 1 [file Presentation1.zip › Supplementary_Material Figures/Fig. S18B Relative abundance of species at genus level for NAKDCAL and NAKDNCAL.jpg]

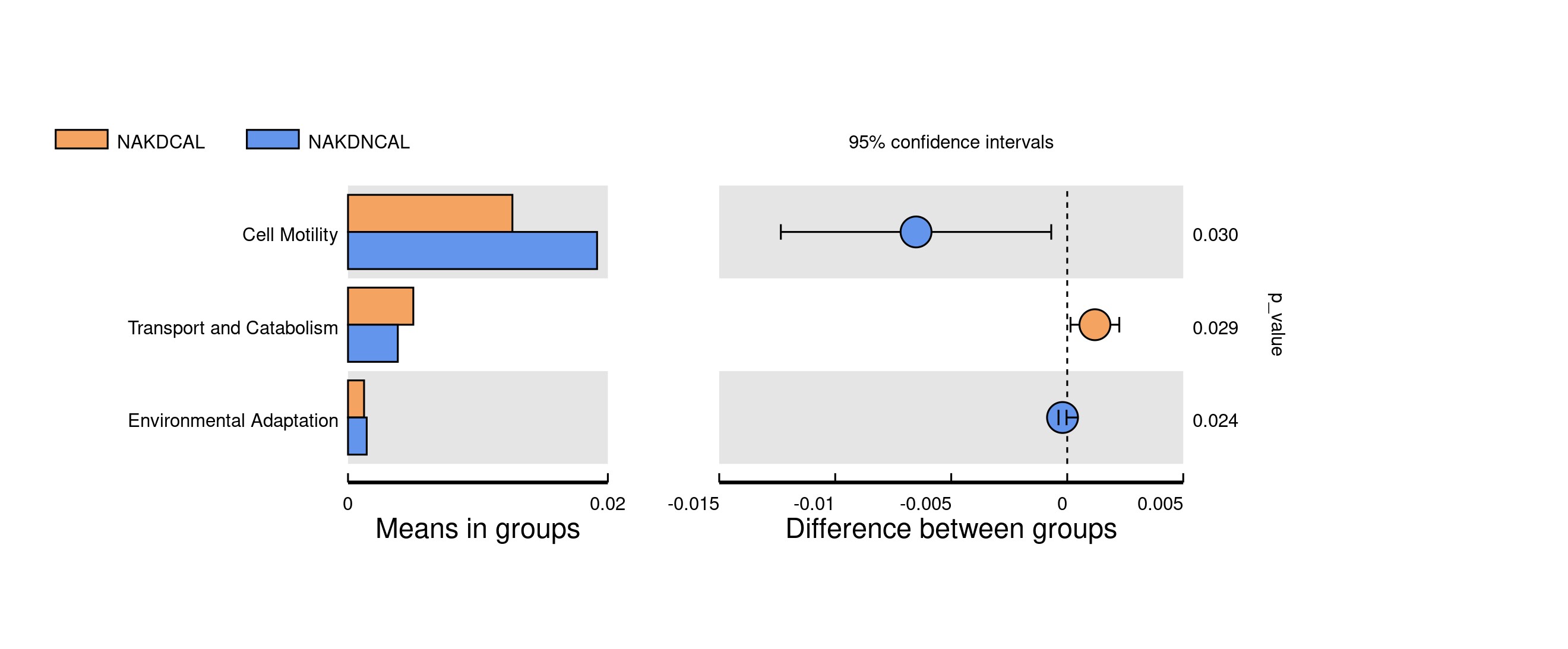

Supplement: Supplementary file 1 [file Presentation1.zip › Supplementary_Material Figures/Fig. S19A Functional capability analysis based on the KEGG pathways at level 2 between NAKDCAL and NAKDNCAL.jpg]

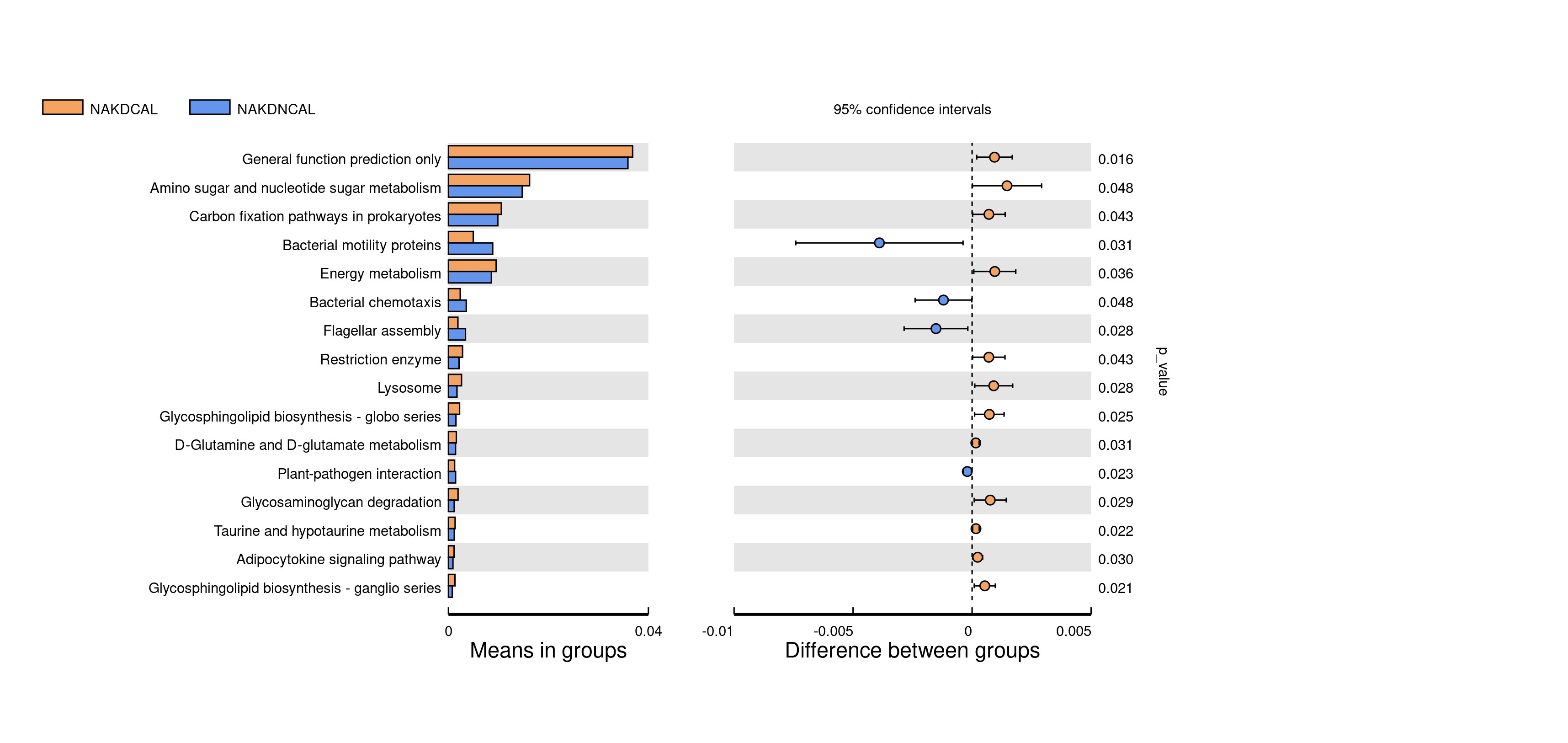

Supplement: Supplementary file 1 [file Presentation1.zip › Supplementary_Material Figures/Fig. S19B Functional capability analysis based on the KEGG pathways at level 3 between NAKDCAL and NAKDNCAL.jpg]

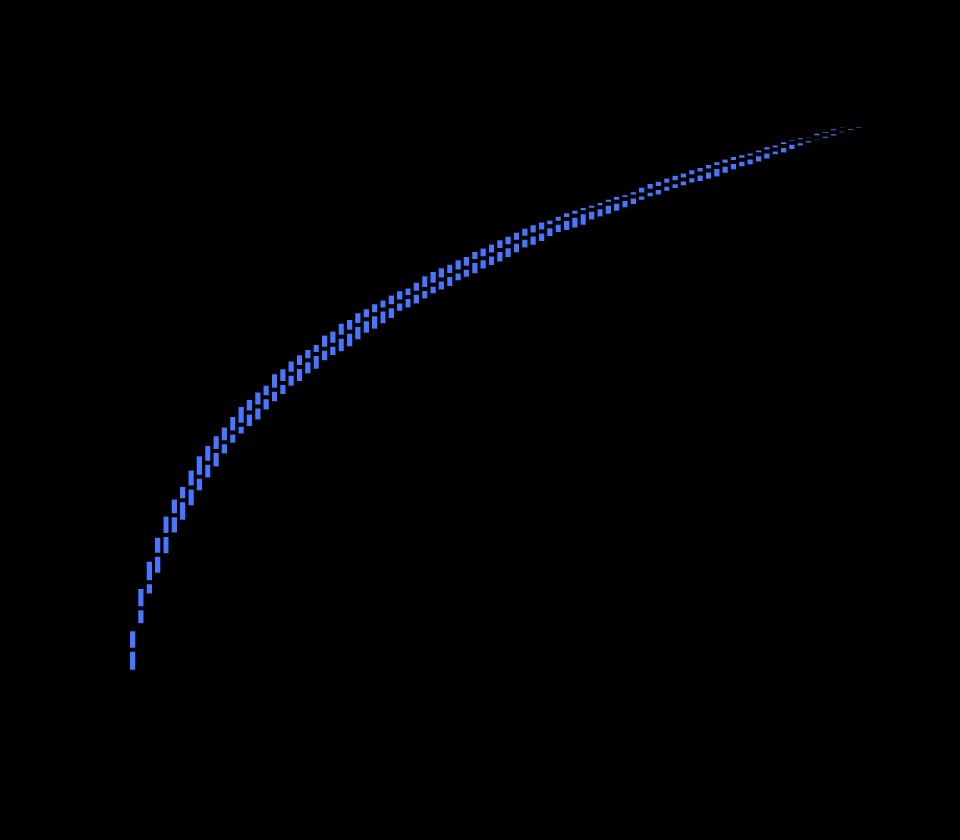

Supplement: Supplementary file 1 [file Presentation1.zip › Supplementary_Material Figures/Fig. S2 Species accumulation curves of all sample.jpg]

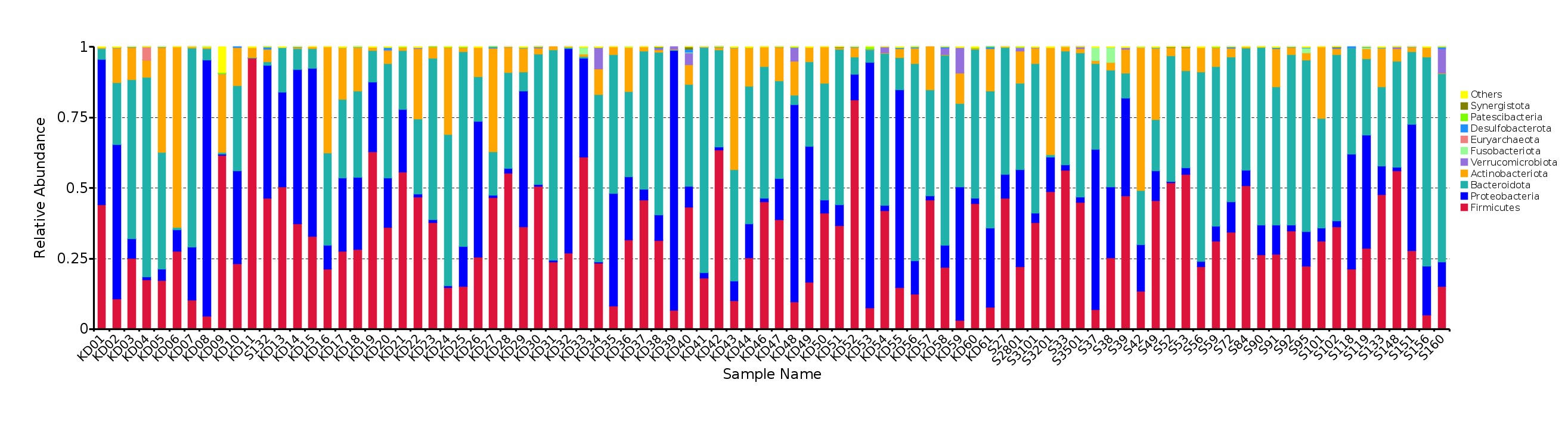

Supplement: Supplementary file 1 [file Presentation1.zip › Supplementary_Material Figures/Fig. S3 Bar plot of relative abundance of species at phylum level for all samples.jpg]

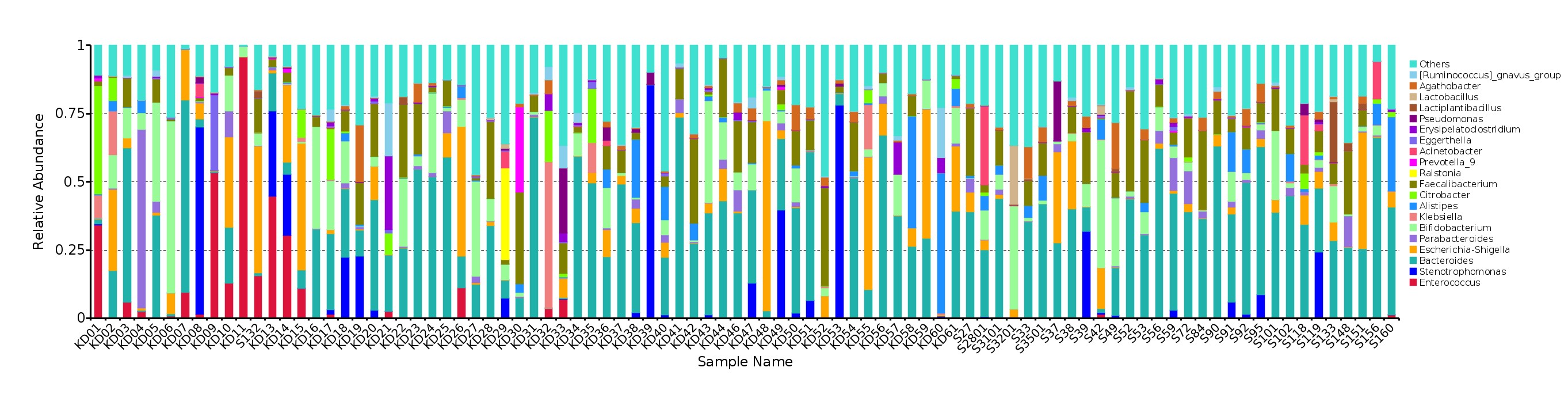

Supplement: Supplementary file 1 [file Presentation1.zip › Supplementary_Material Figures/Fig. S4 Bar plot of relative abundance of species at genus level for all samples.jpg]

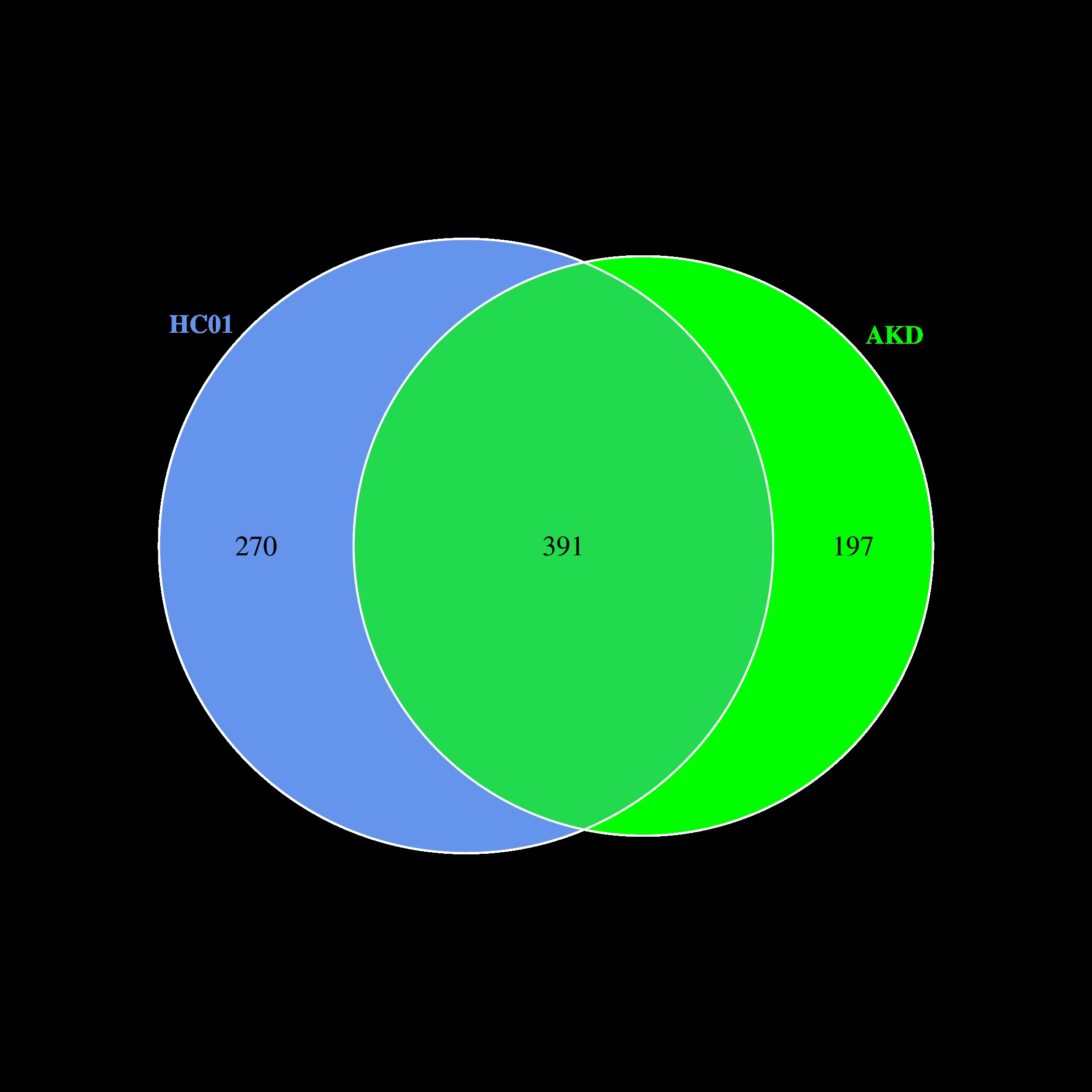

Supplement: Supplementary file 1 [file Presentation1.zip › Supplementary_Material Figures/Fig. S5 Veen diagram of AKD and HC01.jpg]

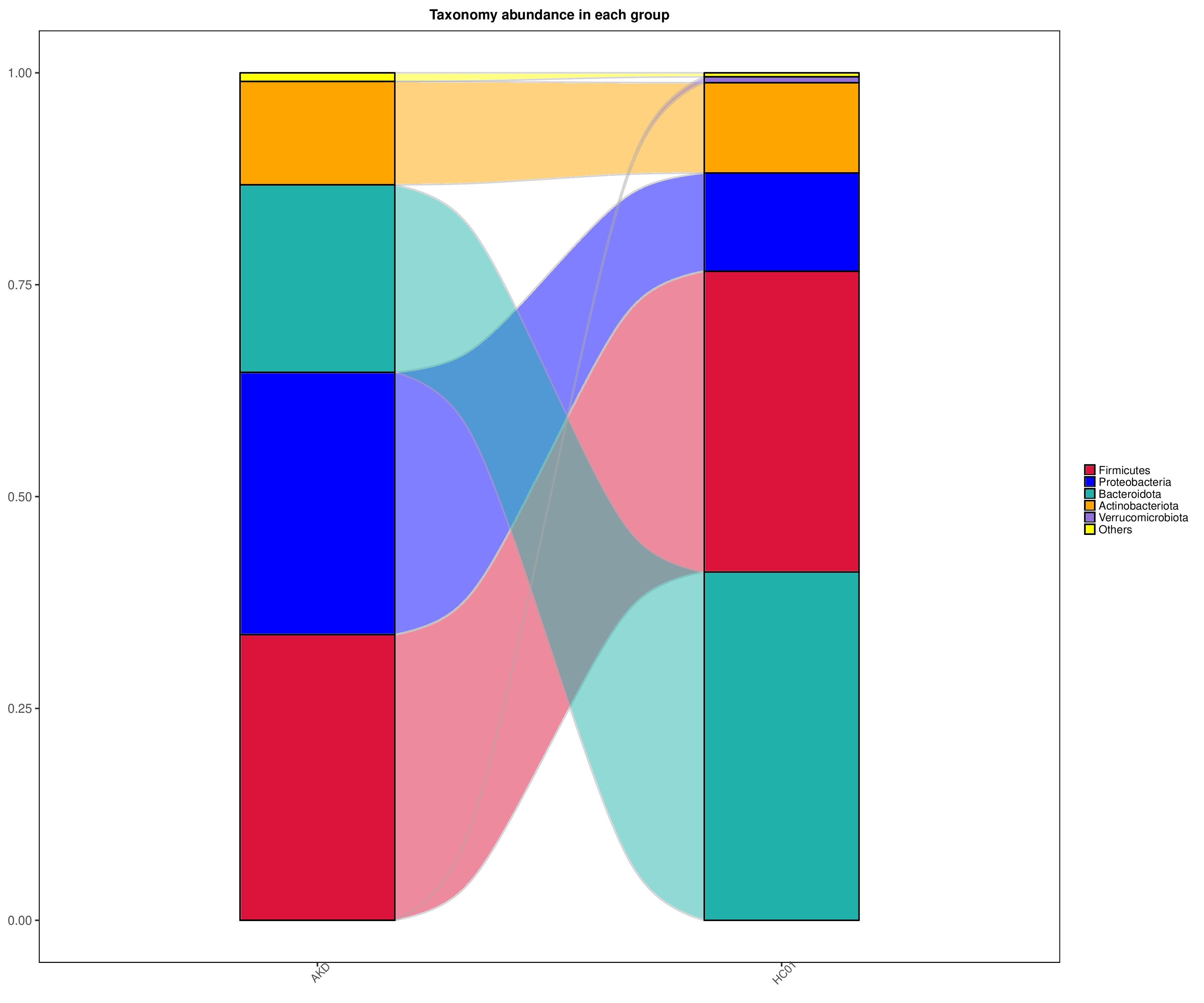

Supplement: Supplementary file 1 [file Presentation1.zip › Supplementary_Material Figures/Fig. S6A Sankey diagram at phylum level between AKD and HC01.jpg]

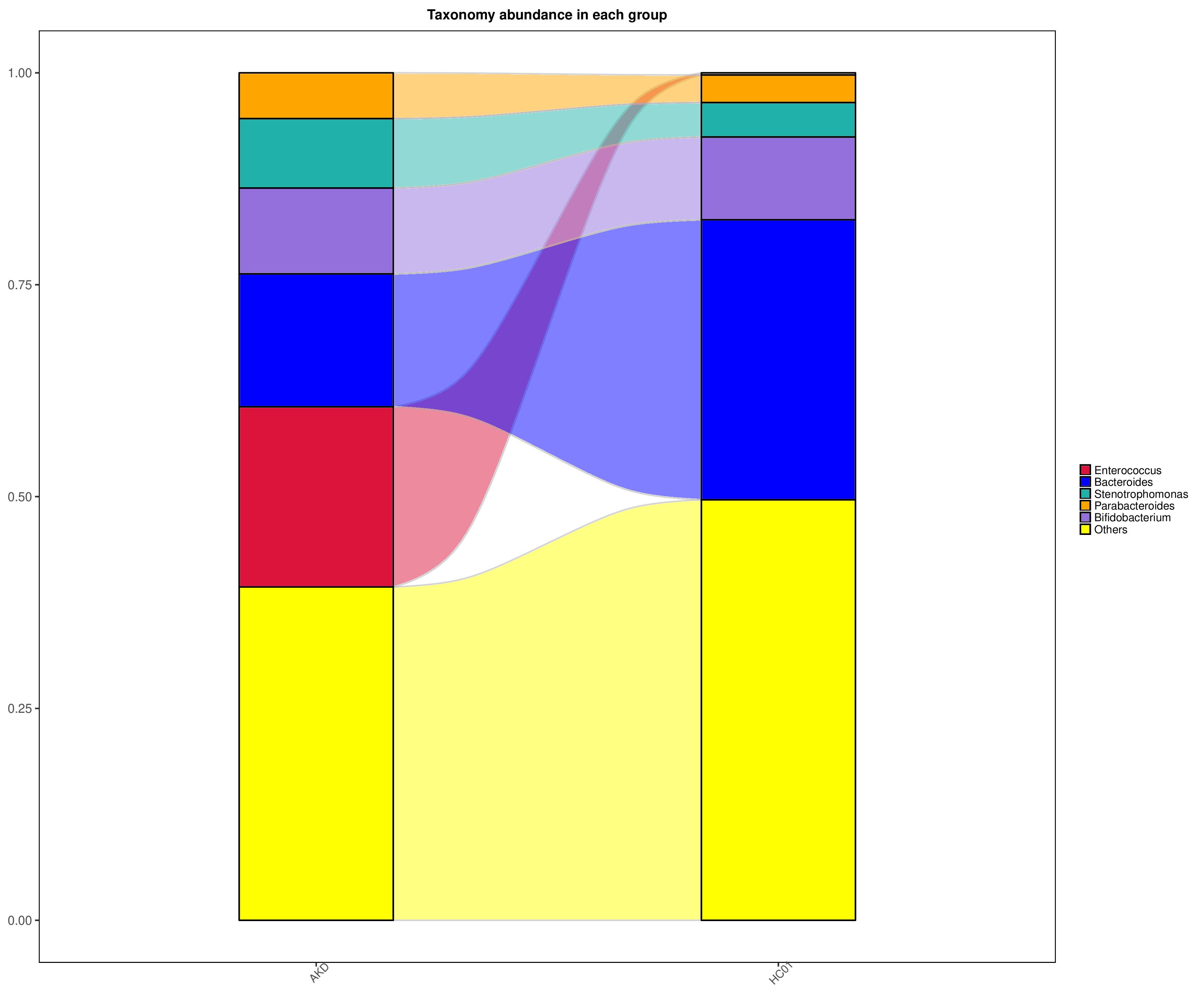

Supplement: Supplementary file 1 [file Presentation1.zip › Supplementary_Material Figures/Fig. S6B Sankey diagram at genus level between AKD and HC01.jpg]

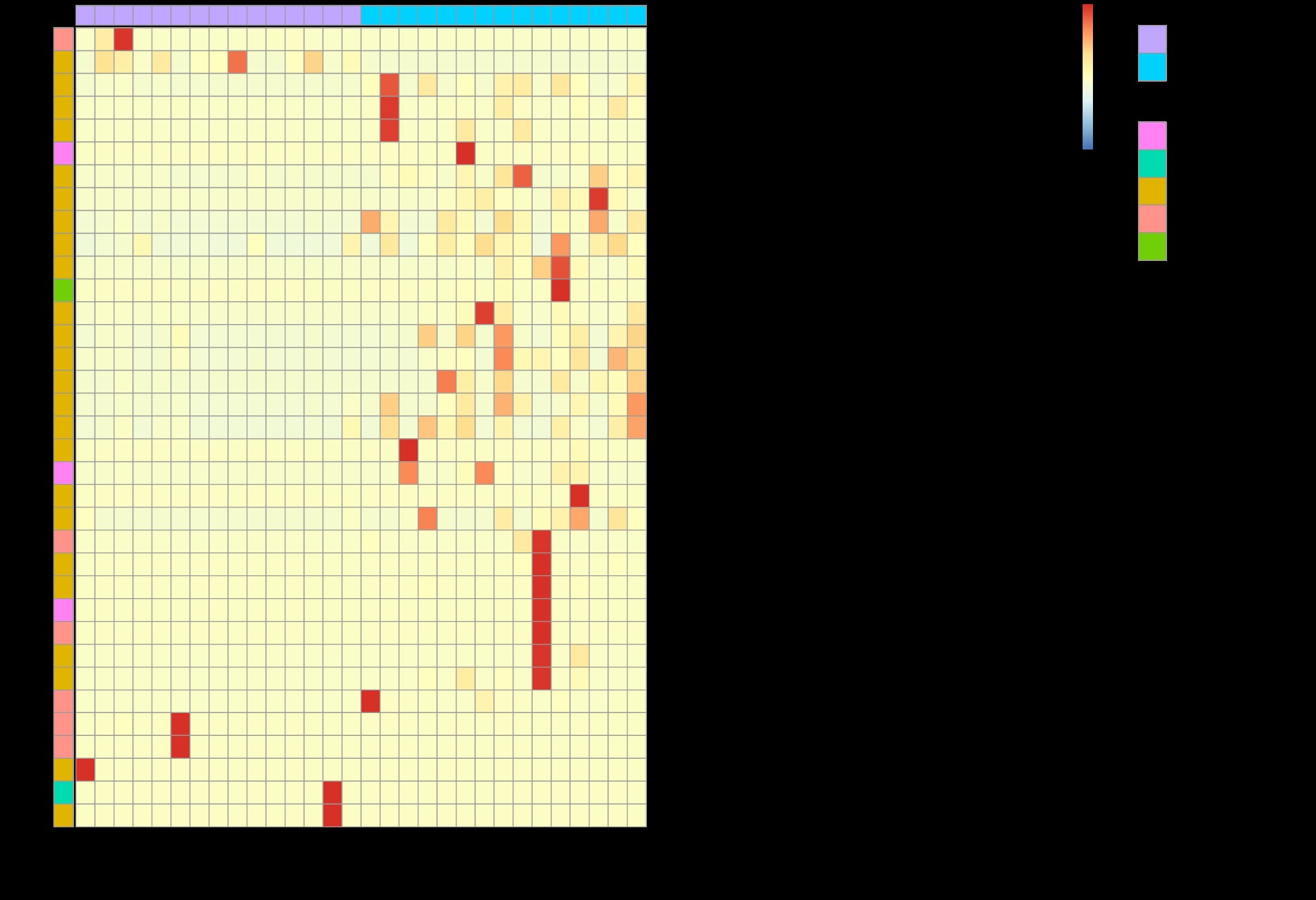

Supplement: Supplementary file 1 [file Presentation1.zip › Supplementary_Material Figures/Fig. S7 Cluster map of meta stat analysis between AKD and HC01.jpg]

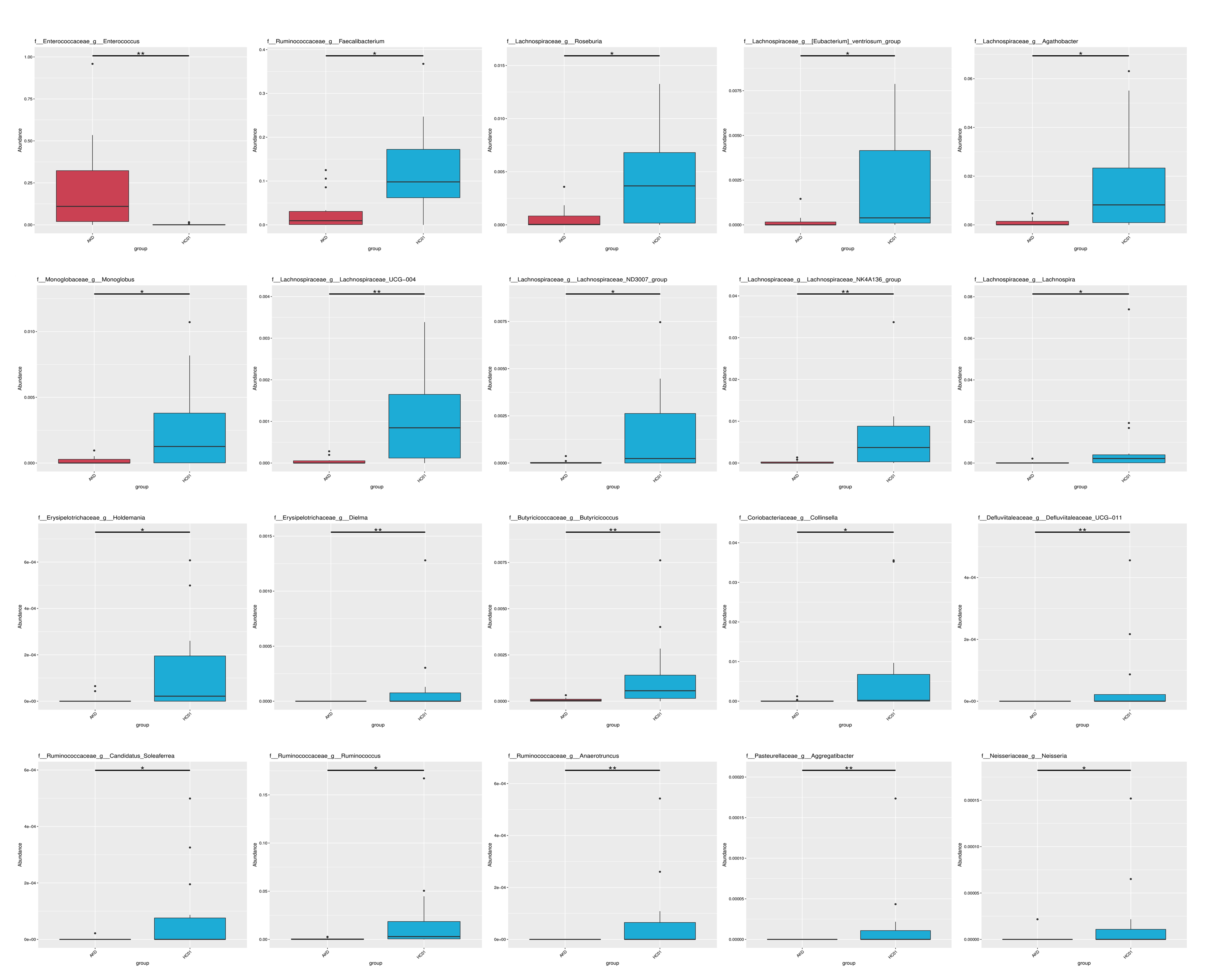

Supplement: Supplementary file 1 [file Presentation1.zip › Supplementary_Material Figures/Fig. S8 Box-plot of meta stat analysis between AKD and HC01.jpg]

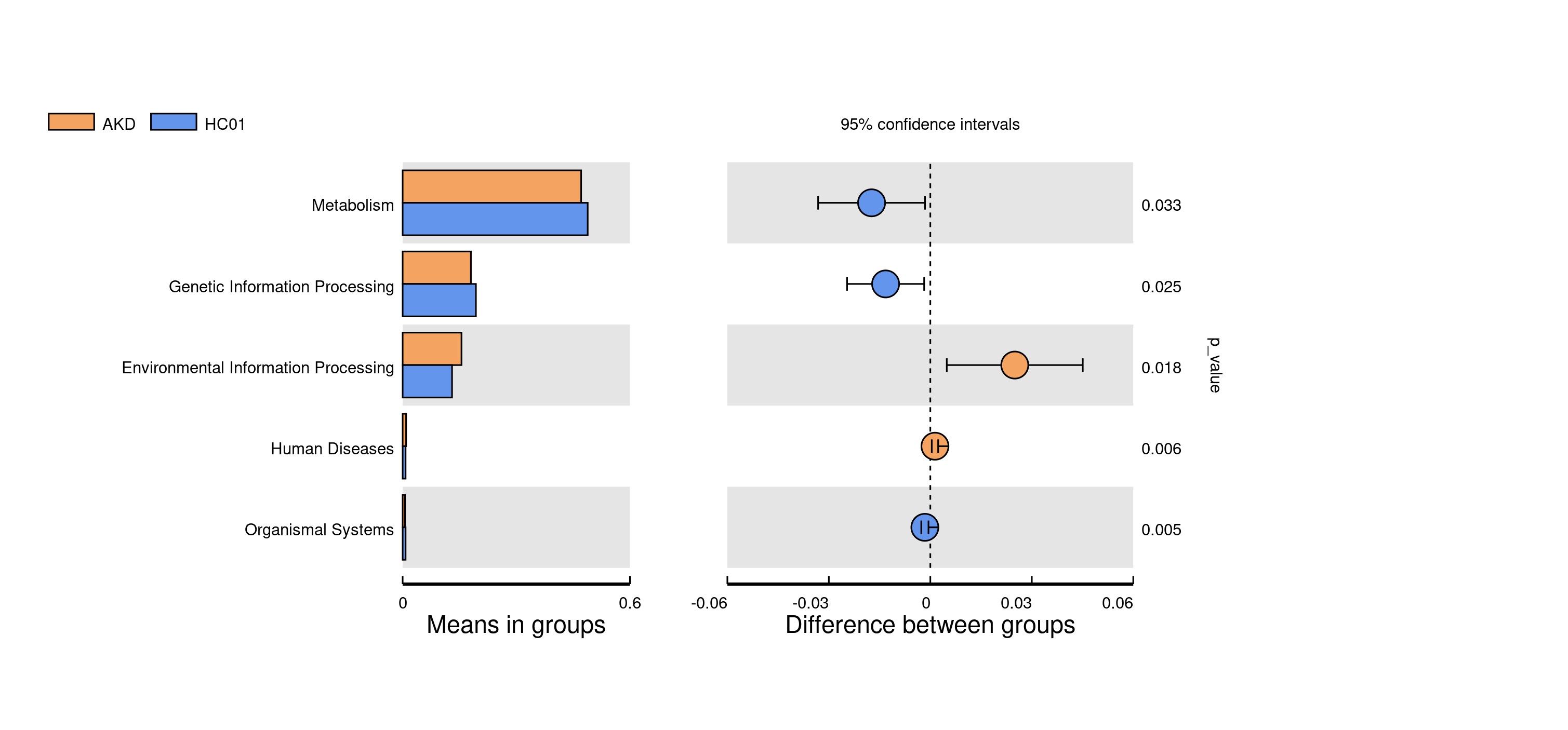

Supplement: Supplementary file 1 [file Presentation1.zip › Supplementary_Material Figures/Fig. S9A Functional capability analysis based on the KEGG pathways at level 1 between AKD and HC01.jpg]

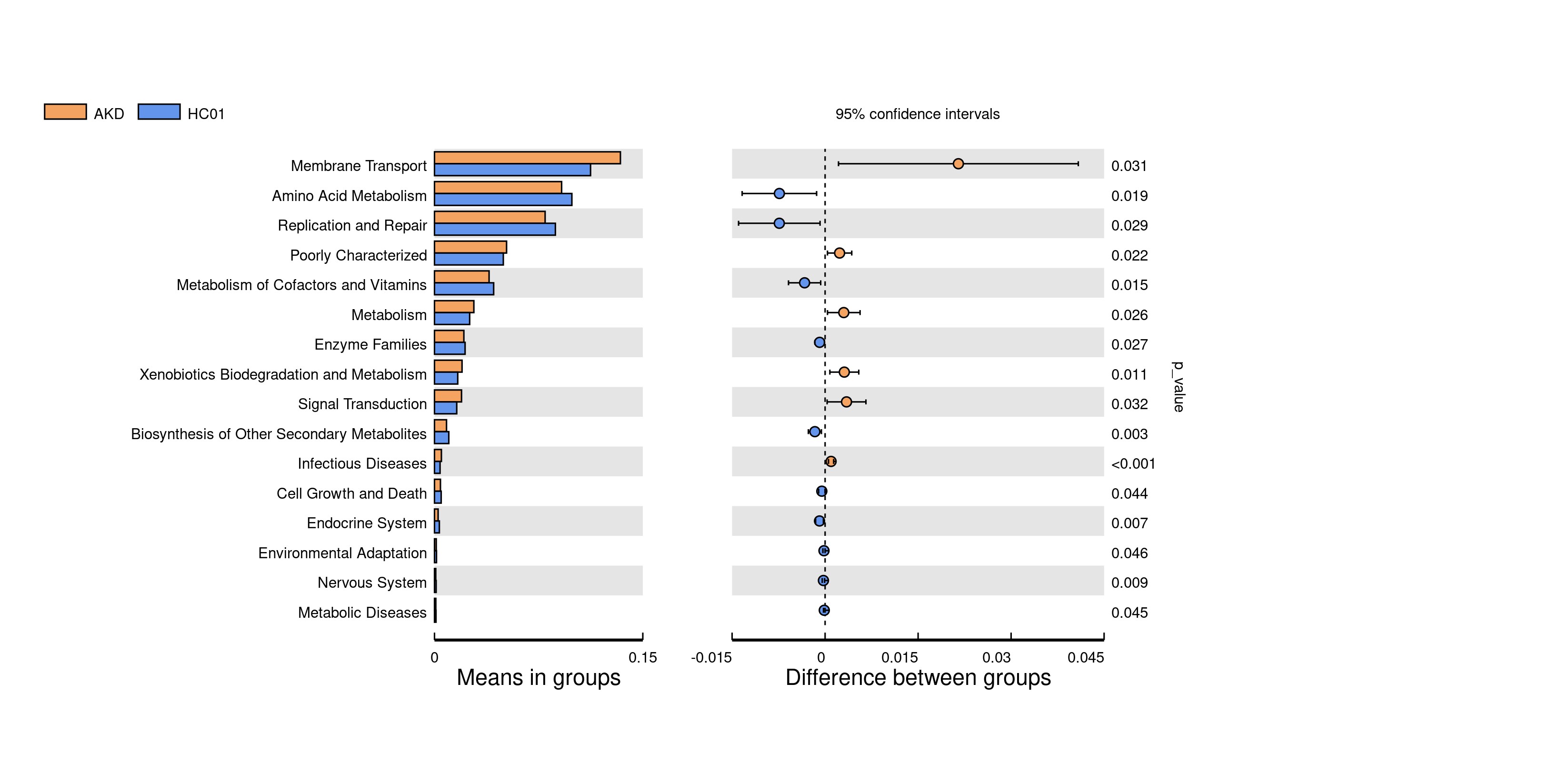

Supplement: Supplementary file 1 [file Presentation1.zip › Supplementary_Material Figures/Fig. S9B Functional capability analysis based on the KEGG pathways at level 2 between AKD and HC01.jpg]

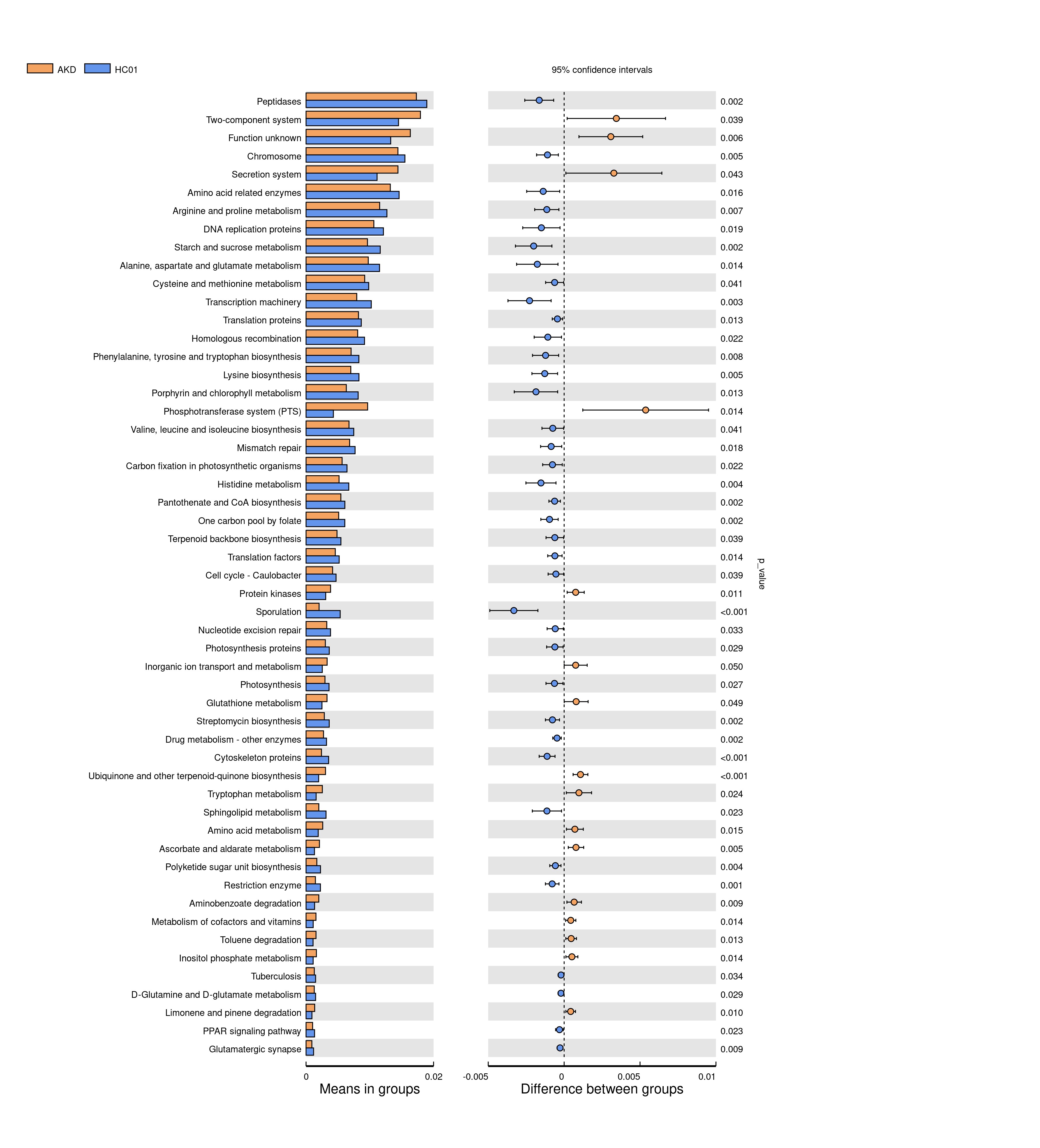

Supplement: Supplementary file 1 [file Presentation1.zip › Supplementary_Material Figures/Fig. S9C Functional capability analysis based on the KEGG pathways at level 3 between AKD and HC01.jpg]
