# Supplementary material for: Unveiling the gut-heart potential connection: microbiota’s role in kawasaki disease and coronary artery lesions
Source: Front Cell Infect Microbiol. 2025 May 29;15:1560083. doi: 10.3389/fcimb.2025.1560083 (PMC12158935; doi:10.3389/fcimb.2025.1560083)
Supplement: Supplementary file 2 [file Table1.docx]

Supplementary Material

# Supplementary Tables

Table 1 The basic information of all KD patients

| **Number** | **Group** | **Age (years)** | **Gender** | **CAL** | **Aspirin** | **Diarrhea** | **Antibiotic** |
| --- | --- | --- | --- | --- | --- | --- | --- |
| KD01 | AKD | 1.50 | Male | No | Yes | Yes | Piperacillin |
| KD02 | AKD | 0.92 | Male | No | Yes | No | Azithromycin |
| KD03 | AKD | 1.50 | Male | Yes | Yes | No | Piperacillin |
| KD04 | AKD | 6.08 | Male | Yes | Yes | No | Cefmetazole |
| KD05 | AKD | 2.08 | Male | Yes | Yes | No | Cefoperazone |
| KD06 | AKD | 5.75 | Male | Yes | Yes | No | Ceftriaxone |
| KD07 | AKD | 1.75 | Male | Yes | Yes | No | Ampicillin |
| KD08 | AKD | 1.08 | Female | No | Yes | No | Cefixime |
| KD09 | AKD | 1.08 | Female | No | Yes | No | Cefixime |
| KD10 | AKD | 1.58 | Female | No | Yes | No | Ceftriaxone |
| KD11 | AKD | 1.58 | Female | No | Yes | Yes | Ceftriaxone |
| S132 | AKD | 4.08 | Female | No | Yes | No | Cefazoxime |
| KD13 | AKD | 2.17 | Female | Yes | Yes | Yes | Cefoperazone |
| KD14 | AKD | 2.17 | Female | Yes | Yes | Yes | Cefoperazone |
| KD15 | AKD | 3.00 | Female | Yes | Yes | No | Erythromycin |
| KD16 | NAKD1 | 4.42 | Male | No | No | No | No |
| KD17 | NAKD2 | 2.00 | Male | No | Yes | No | No |
| KD18 | NAKD1 | 6.00 | Male | No | No | No | No |
| KD19 | NAKD1 | 3.00 | Male | No | No | No | No |
| KD20 | NAKD1 | 3.75 | Male | No | No | No | No |
| KD21 | NAKD2 | 1.33 | Male | No | Yes | No | No |
| KD22 | NAKD1 | 2.50 | Male | No | No | No | No |
| KD23 | NAKD2 | 1.58 | Male | No | Yes | No | No |
| KD24 | NAKD1 | 1.75 | Male | No | No | No | No |
| KD25 | NAKD1 | 3.42 | Male | No | No | No | No |
| KD26 | NAKD1 | 2.00 | Male | No | No | No | No |
| KD27 | NAKD1 | 4.50 | Male | No | No | No | No |
| KD28 | NAKD1 | 1.33 | Male | No | No | No | No |
| KD29 | NAKD1 | 5.00 | Male | No | No | No | No |
| KD30 | NAKD1 | 4.00 | Male | No | No | No | No |
| KD31 | NAKD1 | 5.58 | Male | No | No | No | No |
| KD32 | NAKD2 | 0.75 | Male | No | Yes | No | No |
| KD33 | NAKD2 | 1.50 | Male | Yes | Yes | No | No |
| KD34 | NAKD2 | 2.08 | Male | Yes | Yes | No | No |
| KD35 | NAKD2 | 4.00 | Male | Yes | Yes | No | No |
| KD36 | NAKD1 | 2.00 | Male | Yes | Yes | No | No |
| KD37 | NAKD1 | 2.67 | Male | Yes | Yes | No | No |
| KD38 | NAKD1 | 5.00 | Male | Yes | Yes | No | No |
| KD39 | NAKD1 | 3.42 | Male | No | No | No | No |
| KD40 | NAKD1 | 2.00 | Male | Yes | Yes | No | No |
| KD41 | NAKD1 | 2.08 | Male | Yes | Yes | No | No |
| KD42 | NAKD2 | 3.00 | Male | Yes | Yes | No | No |
| KD43 | NAKD1 | 2.00 | Male | Yes | Yes | No | No |
| KD44 | NAKD1 | 3.92 | Male | Yes | Yes | No | No |
| KD46 | NAKD1 | 6.33 | Female | No | No | No | No |
| KD47 | NAKD2 | 1.83 | Female | No | Yes | No | No |
| KD48 | NAKD2 | 2.17 | Female | No | Yes | No | No |
| KD49 | NAKD2 | 0.92 | Female | No | Yes | No | No |
| KD50 | NAKD1 | 6.17 | Female | No | No | No | No |
| KD51 | NAKD1 | 6.00 | Female | No | No | No | No |
| KD52 | NAKD1 | 1.75 | Female | No | No | No | No |
| KD53 | NAKD1 | 3.00 | Female | No | No | No | No |
| KD54 | NAKD2 | 3.00 | Female | No | Yes | No | No |
| KD55 | NAKD1 | 6.67 | Female | No | No | No | No |
| KD56 | NAKD1 | 7.25 | Female | No | No | No | No |
| KD57 | NAKD1 | 1.25 | Female | No | No | No | No |
| KD58 | NAKD1 | 3.00 | Female | No | No | No | No |
| KD59 | NAKD1 | 7.833 | Female | No | No | No | No |
| KD60 | NAKD1 | 1.417 | Female | Yes | Yes | No | No |
| KD61 | NAKD1 | 4.25 | Female | Yes | Yes | No | No |

Table 2 The basic information of all healthy children

| **Number** | **Age (years)** | **Gender** | **Number** | **Age (years)** | **Gender** |
| --- | --- | --- | --- | --- | --- |
| S27 | 3.75 | Male | S72 | 3.58 | Female |
| S28 | 4.00 | Female | S84 | 7.17 | Female |
| S31 | 6.58 | Female | S90 | 5.00 | Male |
| S32 | 1.00 | Female | S91 | 6.00 | Female |
| S33 | 7.58 | Male | S92 | 6.00 | Male |
| S35 | 3.17 | Male | S95 | 6.08 | Female |
| S37 | 4.58 | Male | S101 | 2.25 | Female |
| S38 | 2.58 | Male | S102 | 4.00 | Female |
| S39 | 1.17 | Male | S118 | 4.5 | Female |
| S42 | 0.75 | Female | S119 | 0.42 | Male |
| S49 | 4.83 | Male | S133 | 5.25 | Male |
| S52 | 3.50 | Female | S148 | 1.42 | Female |
| S53 | 4.33 | Female | S151 | 6.75 | Male |
| S56 | 1.75 | Female | S156 | 5.00 | Male |
| S59 | 3.50 | Male | S160 | 2.42 | Female |

Table 3 The basic information of all groups.

| **Group** | **Total** | **CAL** | **NCAL** | **Age (years)** | **Female： Male** |
| --- | --- | --- | --- | --- | --- |
| AKD | 15 | 8 | 7 | 2.42±1.63 | 7:8 |
| NAKD1 | 12 | 4 | 8 | 2.01±0.94 | 8:4 |
| NAKD2 | 33 | 9 | 24 | 3.80±1.88 | 21:12 |
| HC01 | 15 | \ | \ | 2.48±1.47 | 7:8 |
| HC01 | 30 | \ | \ | 3.96±1.99 | 14:16 |
